# Diversity, molecular identification and plant growth promoting potential of endophytic bacteria from plantain (*Plantago lanceolata* L.)

**DOI:** 10.3389/fmicb.2026.1815167

**Published:** 2026-06-08

**Authors:** Dilfuza Jabborova, Bakhora Djalolova, Numonjon Sultanov, Muzafar Jabborov, Nasir Mehmood, Sokhibjon Abdusamatov, Khasan Kayumov, Usman Zulfiqar, Hossam S. El-Beltagi, Abdulrahman Alasmari, Mayank Anand Guruani

**Affiliations:** 1 Institute of Genetics and Plant Experimental Biology of the Academy of Sciences of Uzbekistan, Kibray, Uzbekistan; 2 Faculty of Biology and Ecology, National University of Uzbekistan, Tashkent, Uzbekistan; 3 Division of Plant Biotechnology, Sher-e-Kashmir University of Agricultural Sciences and Technology of Kashmir, Shalimar, Jammu and Kashmir, India; 4 Academy of Biology and Biotechnology, Southern Federal University, Rostov-on-Don, Russia; 5 Department of Agronomy, Faculty of Agriculture and Environment, The Islamia University of Bahawalpur, Bahawalpur, Pakistan; 6 Department of Biology, Nakhchivan State University, Nakhchivan, Azerbaijan; 7 Agricultural Biotechnology Department, College of Agriculture and Food Sciences, King Faisal University, Al-Ahsa, Saudi Arabia; 8 Department of Biology, Faculty of Science, University of Tabuk, Tabuk, Saudi Arabia; 9 Biodiversity Genomics Unit, Faculty of Science, University of Tabuk, Tabuk, Saudi Arabia; 10 Department of Biology, College of Science, United Arab Emirates University, Al Ain, United Arab Emirates

**Keywords:** 16S rRNA gene sequencing, ACC deaminase activity, antifungal activity, endophytic bacteria, phosphate solubilization, plant growth-promoting bacteria, *Plantago lanceolata L*.

## Abstract

*Plantago lanceolata* L. is a perennial medicinal plant widely recognized for its pharmacological importance and ecological adaptability. In addition to its therapeutic value, this species hosts diverse communities of endophytic bacteria that may significantly influence plant growth, metabolic activity, and tolerance to environmental stresses. The present study aimed to isolate, identify, and functionally characterize cultivable endophytic bacteria associated with the leaves and roots of healthy *Plantago lanceolata* L. plants collected from Surkhandarya State Forestry, Termez district, Surkhandarya region, Uzbekistan. Surface-sterilized plant tissues were aseptically processed, and bacterial endophytes were isolated on nutrient agar (NA). Molecular identification of the isolates was performed using 16S rRNA gene sequencing followed by sequence comparison through NCBI BLAST analysis. Six representative bacterial strains (IGPEB1–IGPEB6) were identified and their nucleotide sequences were deposited in GenBank under the accession numbers PV848383 (*Pseudomonas* sp. strain IGPEB1), PV848384 (*Brevibacillus* sp. strain IGPEB2), PV848385 (*Brevibacillus* sp. strain IGPEB3), PV848386 (*Bacillus* sp. strain IGPEB4), PV848387 (*Bacillus* sp. strain IGPEB5), and PV848388 (*Bacillus* sp. strain IGPEB6). Functional characterization revealed that the isolates possessed multiple plant growth-promoting (PGP) traits, including indole-3-acetic acid (IAA) production, phosphate solubilization, ammonia production, and extracellular enzymatic activities. These findings demonstrate the taxonomic and functional diversity of endophytic bacteria associated with *P. lanceolata* L. and highlight their potential application as biofertilizers and biocontrol agents to support sustainable agricultural productivity. To the best of our knowledge, this study represents the first report describing endophytic bacterial communities associated with *P. lanceolata* L. in the Surkhandarya State Forestry region of Uzbekistan, providing a valuable foundation for future ecological and biotechnological investigations.

## 1 Introduction

*Plantago lanceolata* L. (*Plantaginacea*e) is a perennial medicinal plant widely distributed across Europe, the Mediterranean region, and Central and Western Asia, where it is also reported as an invasive species in certain habitats. This species has long been utilized in traditional medicine, particularly for wound healing, and has been demonstrated to possess anti-inflammatory, analgesic, and antihistamine properties (Samuelsen, 2000; Aremu et al., 2024; Drożdżyński et al., 2024; Zhakipbekov et al., 2023). Recent studies have further revealed that root extracts of *P. lanceolata* L. are rich in bioactive compounds and exhibit notable cytotoxic and antibacterial activities (Rahamouz-Haghighi et al., 2022). These findings highlight the plant's potential not only in pharmaceutical applications but also as a promising resource in agrobiotechnology.

In recent years, interactions between plants and microorganisms have been extensively investigated within the framework of the “plant microbiome” or “holobiont” concept. According to this paradigm, the plant and its associated microbiota are considered as an integrated functional unit that collectively determines plant growth, development, and stress resilience. Endophytic microorganisms represent a key component of this microbiome, residing within internal plant tissues and exerting direct influences on plant physiology (Kirchhoff et al., 2019; Abdusamatov et al., 2025a). Currently, *P. lanceolata* L. is widely employed as a model organism in ecological studies, particularly in investigations of plant–microbe interactions, population ecology, and adaptive responses to diverse environmental conditions (Kirchhoff et al., 2019; Laanet et al., 2024). Notably, endophytic bacteria play a crucial role in enhancing plant stress tolerance, improving nutrient acquisition, and providing protection against phytopathogens (Tang et al., 2025).

However, studies conducted to date have focused mainly on individual plant organs or individual components of the microbiome, which has led to insufficient study of the organ-specific diversity and functional potential of endophytic bacteria in *Plantago lanceolata* L. In addition, information about endophytic bacteria specific to this plant species in Central Asia, especially in Uzbekistan, is extremely limited, which prevents a full assessment of their ecological adaptation and agrobiotechnological application potential. The Surkhandarya region of the Republic of Uzbekistan, where the study was conducted, is one of the extreme agroecological regions characterized by high temperature, drought and soil degradation. The plant-microorganism system formed under such conditions, in particular endophytic bacteria, may have a high level of stress adaptability and functional activity (Hardoim et al., 2008; Santoyo et al., 2016; Afzal et al., 2019; Khasanov et al., 2023). Therefore, microorganisms isolated from this region are of significant scientific and practical importance in the development of sustainable agriculture and environmentally friendly biotechnology. Endophytic bacteria promote plant growth through multiple mechanisms, including the production of indole-3-acetic acid (IAA), phosphate solubilization, siderophore biosynthesis, and ACC deaminase activity (Panigrahi et al., 2020; de Lima et al., 2024; Biswas et al., 2023; Younis et al., 2024; Djalolova et al., 2024; Ramírez et al., 2024). These mechanisms collectively enhance root and shoot development, increase nutrient uptake efficiency, and improve plant tolerance to abiotic stresses (Kaur and Karnwal, 2023; Corral-Federico et al., 2024). Therefore, the application of endophytic bacteria as bioinoculants represents a key strategy for advancing sustainable and environmentally friendly agriculture (de Lima et al., 2024; Djalolova et al., 2024; Sherzad et al., 2025; Jabborova et al., 2025a; Abdusamatov et al., 2025b). In parallel, the microbiota of this plant, particularly its endophytic bacterial community, has attracted increasing scientific attention. Previous studies have reported the isolation of several endophytic bacteria from the roots of *P. lanceolata* L., including *Bacillus methylotropicus* and *Pseudomonas brassicacearum*, while other isolates were assigned to the genus *Bacillus*. These bacteria exhibited strong biocontrol activity against several phytopathogens, including *Agrobacterium* spp. and *Pectobacterium* spp (Krimi et al., 2016). Furthermore, Souza et al. (2024) demonstrated that seeds of *Plantago lanceolata* L. harbor a stable core microbiome that is maintained regardless of plant diversity levels. This core microbiome predominantly consists of endophytic bacteria belonging to the genera *Pseudomonas, Sphingomonas*, and *Pirellula*, which play significant roles in plant development and adaptation. These findings further confirm the complexity and functional importance of the microbiome associated with this species.

At the same time, existing studies have largely focused on specific plant organs or individual components of the microbiome (Penczykowski et al., 2016; Abdusamatov et al., 2024), while the diversity and functional characteristics of endophytic bacteria associated with different vegetative organs of *P. lanceolata* L. remain insufficiently explored. In particular, data on the microbiome associated with this species under Central Asian conditions are very limited (Souza et al., 2024). Therefore, comprehensive investigation of plant-associated microbiomes and their functional composition represents a critical and timely direction in modern microbiology and agrobiotechnology. From this perspective, the present study aimed to isolate endophytic bacteria from the roots, stems, and leaves of *P. lanceolata* L. growing in the Surkhandarya region of Uzbekistan. The isolates were subsequently characterized using both morphological and molecular approaches, including 16S rRNA gene-based identification. In addition, the plant growth-promoting traits of the isolates were evaluated, including phytohormone production, phosphate and zinc solubilization, siderophore biosynthesis, and enzymatic activities.

## 2 Materials and methods

### 2.1 Sampling site and sample collection

Plant samples for the study were collected from the territory of the Surkhandarya State Forestry Enterprise, located in Termez district of the Surkhandarya region of the Republic of Uzbekistan (N 37°13′44″, E 67°16′34″). The sampling coordinates are. Sampling was carried out in the first 10 days of May 2025. This area is characterized by arid climatic conditions and is distinguished by a variety of medicinal plants. Five independent and healthy *P. lanceolata* L. plants, similar in morphological characteristics (plant height, number of leaves, and growth stage), were selected and accepted as biological replicates. Each plant was carefully uprooted, and root, stem, and leaf tissues were collected separately to ensure representative sampling. After collection, the plant samples were transported to the laboratory for further processing and stored in labeled zip-bags with ice packs to preserve sample integrity. All samples were processed within 24 h after collection to maintain microbial viability.

### 2.2 Surface sterilization and isolation of endophytic bacteria

The protocol described by McKinnon (2016) was followed to remove epiphytes and non-endophytic microorganisms. In order to get rid of undesirable soil particles, samples were carefully cleaned using tap water. Root, stem, and leaf tissues were processed separately for the isolation of endophytic bacteria. The tissues were cut into approximately 2–3 cm long pieces using a sterilized scalpel, placed in a permeable container and washed in a 0.05 % Tween-80 solution. After disinfection with 70% ethanol for 1 min, the samples were immersed in a 2.5% sodium hypochlorite solution (Sigma-Aldrich, USA) for 4 min. Samples were washed again with 70% ethanol for 30 s. Finally, the samples were thoroughly rinsed twice with sterile distilled water (1 min each). Following sterilization, the tissues were kept at −20°C for further analysis after being air dried in a laminar flow cabinet. A 100 μL aliquot of the final rinse water was plated on tryptone soy agar (TSA) and incubated at 30°C for 3–5 days to confirm the effectiveness of surface sterilization. The absence of microbial growth on the medium confirmed the effectiveness of surface sterilization.

Sterilized plant tissues were grounded in a laminar flow hood using a sterile mortar and pestle to form a suspension. 1 g of the crushed tissue was taken, mixed with 9 mL of sterile saline (0.9% NaCl), and tenfold serial dilutions were prepared from 10^−1^ to 10^−8^. A100 μL suspension of each dilution was inoculated on TSA or nutrient agar. Sterilized 2–3 cm tissues were also placed on the plates and incubated at 28 ± 2 °C for 48–72 h. The colonies were counted and expressed as CFU g^−1^. All experiments were conducted in triplicate, and results were expressed as mean ± standard deviation. Morphologically distinct colonies were picked, transferred to Tryptone Soy Broth medium in McCartney tubes, and re-plated to obtain pure colonies (Abbas et al., 2024). All procedures were carried out under aseptic conditions to prevent contamination.

### 2.3 *In vitro* germination assay of *Plantago lanceolata* seeds

The plant growth–promoting activity of endophytic bacterial isolates was evaluated by assessing the germination of *Plantago lanceolata* L. seeds under *in vitro* conditions. Seeds of *P. lanceolata* L. were surface sterilized using 5% sodium hypochlorite (Sigma-Aldrich, USA) for 5 min and subsequently rinsed six times with sterile distilled water to remove any residual disinfectant.

Sterilized seeds were placed on Petri dishes containing half-strength Murashige and Skoog ($\frac{1}{2}$ MS) medium (Duchefa Biochemie, Haarlem, The Netherlands) supplemented with 0.5% (w/v) sucrose and solidified with 0.8% (w/v) bacteriological agar. The plates were kept at 4 °C for 24 h for stratification and then transferred to a growth chamber under controlled conditions (16 h light/8 h dark photoperiod at 22 °C).

Endophytic bacterial isolates were cultured on nutrient agar (NA) and a single colony was transferred into nutrient broth (NB) and incubated overnight at 28 °C with shaking at 180 rpm. The bacterial suspension was adjusted to approximately 108 CFU mL^−1^. An aliquot of 10 μL of the bacterial suspension was applied to the seeds on the agar medium. Seeds treated with sterile distilled water served as the control (Thomloudi et al., 2021).

Seed germination was monitored daily, and the germination percentage was recorded on the 4th and 7th days after inoculation. Each treatment consisted of three replicates.

### 2.4 *In vitro* antifungal activity of endophytic bacteria

All isolated bacterial strained undergone *in vitro* double-culture tests. The test was performed on Waxman agar adapted for mold growth. After sterilization, the following substances were added: KH_2_PO_4_ (3.92 μM), NaCl (4.02 μM), KCl (1.8 μM), CaCl_2_ (100 μM), MgCl_2_ (134 μM), H_3_BO_3_ (5 μM), L-ascorbic acid (850 μM), Al_2_(SO_4_)_3_ (0.95 μM), K_2_SO_4_ (0.95 μM), and CuSO_4_ (0.25 μM). The antifungal activity of the isolated endophytic bacteria was tested against *Fusarium solani*. Mycelial fragments from the periphery of an active fungal culture were placed in the middle of a Petri dish to measure the antagonistic effect. At equal distances from the fungal inoculum, four bacterial isolates were inoculated in stripes. Bacterial-free fungal plates served as controls. For 72 hours, the plates were incubated at 28°C and the experiment was replicated thrice (Lovecká et al., 2023; Abdusamatov et al., 2025a,b; Sriwati et al., 2023). Antagonistic activity was evaluated by measuring the inhibition zone between the bacterial streak and the fungal colony. The inhibition distance was measured in millimeters (mm).

In addition, the percentage of mycelial growth inhibition was calculated using the following formula:

Inhibition (%) = [(C – T)/C] × 100,

where *C* is the radial growth of the fungus in the control plate and *T* is the radial growth in the presence of bacterial isolates.

### 2.5 DNA extraction, PCR fingerprinting and molecular identification of endophytic bacteria

Endophytic bacteria were incubated on TSA medium at 37 °C for 24 h. A small portion of bacterial colonies was transferred to a sterile 1.5 mL microcentrifuge tube. DNA was isolated using the Ezup column bacterial genomic DNA isolation kit according to the manufacturer's instructions. The obtained DNA was stored at −20°C.

A partial fragment of the 16S rRNA gene of the isolated DNA was amplified by polymerase chain reaction (PCR) using universal primers 27F (5′-AGAGTTTGATCCTGGCTCAG-3′) and 1492R (5′-TACGGYTACCTTGTTACGACTT-3′) (Lane, 1991). The PCR reaction mixture (25 μL) contained 12.5 μL of OneTaq 2 × Master Mix, 50 ng of DNA template, each primer at a concentration of 10 μM, and sterile nuclease-free water to complete the final volume. Amplification was performed in a Bio-Rad T100 thermocycler under the following conditions: initial denaturation at 94 °C for 5 min, followed by 35 cycles of denaturation at 94°C for 30 s, annealing at 55°C for 35 s, and extension at 72 °C for 1 min, with a final extension at 72 °C for 8 min. The PCR products were verified by electrophoresis on 1% agarose gel. The amplified products were purified using a GeneJET PCR Purification Kit (Thermo Scientific, USA) and prepared for sequencing using the BigDye Terminator v3.1 Cycle Sequencing Kit (Applied Biosystems, USA) (Vanhee et al., 2024).

The obtained sequences were analyzed using BLAST (Basic Local Alignment Search Tool) in the NCBI database for molecular identification of the isolates. Sequence alignment was performed using the MUSCLE algorithm, and a phylogenetic tree was constructed using the neighbor-joining method. Evolutionary distances were calculated using the maximum composite likelihood model implemented in MEGA X software.

### 2.6 Detection of hydrogen cyanide (HCN) production by endophytic bacteria

The assessment of HCN production was conducted by inoculating bacterial suspension in King's B broth medium supplemented with glycine (4.4 g L^−1^). Uninoculated flasks served as controls. Sterile filter paper pieces were dipped in an alkaline picrate suspension (0.5% picric acid and 2% Na_2_CO3) and tied to the neck of the flask (one per flask). The capped and sealed flasks were incubated at 140 rpm for 4 days at 28 °C. The experiment was replicated thrice.

The sodium picrate lines changed color to reddish in proportion to the amount of HCN produced. Fresh filter paper was inserted into 10 mL deionized water test tube and the color absorbance were measured at 625 nm using a spectrophotometer (SHIMADZU, model number UV1800) (Abd El-Rahman et al., 2019).

### 2.7 Detection of siderophore production

Endophytic bacterial colonies were grown in LB broth for 48 h and used for quantitative siderophore production. A 1.5 mL centrifuge tube was sterilized prior to inoculation with 10 μL of fresh bacterial culture (108 CFU/mL), and 1 mL of broth from each bacterial and fungal culture (one for each) was added. For every strain, three replicates (tubes) were employed. A control tube containing uninoculated broth was also made. After 48 hours of incubation at 28°C, endophytic isolate cultures were centrifuged for 10 min at 10,000 rpm, and the siderophore levels were measured in the supernatant. A spectrophotometer (SHIMADZU, UV1800) was used to measure the optical density at 630 nm after 20 min of mixing 0.5 mL of each culture's supernatant with 0.5 mL of CAS mixture (Sarvepalli et al., 2023).

The following formula was used to estimate the percentage (%) siderophore units (psu) of siderophore produced by the strains:

Percent siderophore unit = $\frac{Ar-As}{Ar}\times \;100$

where Ar is the control absorbance at 630 nm (CAS solution and uninoculated broth), As is the sample absorbance (CAS solution and sample supernatant).

### 2.8 Phosphate and zinc solubilization assay

To determine the solubility of phosphorus (P) and zinc (Zn), Pikovskaya agar with 0.5 % Ca3(PO4)_2_ and mineral salt agar with 0.1 % ZnO were used. Bacterial isolates were initially incubated on nitrogen-free (NF) agar. A sterile cork borer was used to create 6 mm diameter agar disks once the isolates had been cultured on the surface of this medium for 24 h. After being moved to indicator media, these disks were incubated for 1 day at 30 °C to determine their Zn solubility and seven days to determine their P solubility. The solubilizing activity of the bacterial strain was demonstrated by the halozones that developed around the agar during the incubation time (Gupta et al., 2024).

### 2.9 Determination of ACC deaminase activity

A modified variant of the Penrose and Glick (2003) method was used to undertake quantitative study of ACC deaminase activity. Measuring the amount of α-ketobutyrate (α-KB) produced through ACC hydrolysis is the basis of this technique. TSB (Tryptic Soy Broth) medium was used to cultivate endophytic bacterial cells at 28 °C. The bacteria were grown overnight, centrifuged for 10 min at 10,000 × g, and then rinsed with 0.1 M Tris–HCl (pH 7.5) before being suspended in DF medium with 3 mM ACC as the only nitrogen source. After 48 h of incubation at 28°C, the biomass was extracted using centrifugation at 10,000 × g for 10 min at 4 °C.

Five milliliters of DF minimal salt media were used to extract and wash the cells in the supernatant twice. After that, the cells were moved to a fresh tube, suspended in 7.5 mL of DF low salt medium that had been supplemented with 3 mM ACC, and shaken at 140 rpm/min for 24 h. at 28 °C. After centrifuging the induced cells for 10 min at 6,000 × g, they were rinsed with 0.1 M Tris-HCl (pH 7.6). The pellets were then resuspended in 200 μL of 0.1 mol L^−1^ Tris-HCl (pH 8.5) after the tubes had been centrifuged for 5 min at 12,000 × g. After adding 10 μL of toluene, the cleaned cells were labialized and vortexed for 30 s at maximum speed. Then, 50 μL of the toluene-treated cell suspension was transferred to a new tube, 5 μL of 0.5 M ACC was added, and incubated at 30 °C for 15 min. 250 μL of the supernatant was removed, 200 μL of 0.56 N HCl and 75 μL of 2,4-DNF (0.2% 2,4-dinitrophenylhydrazine in 2 mol L^−1^ HCl) were added, and the mixture was incubated at 30 °C for 30 min. Then, 500 μL of NaOH (2N) was added, and the absorbance of the resulting mixture at 540 nm was measured using a Shimadzu UV-1,800 spectrophotometer.

The negative control was a cell suspension devoid of ACC positive control was the medium with (NH_4_)_2_SO_4_. The α-ketobutyrate concentration recorded using standard calibration curve ranging from 0.1 to 1 mM. ACC deaminase activity was calculated based on the production of μmol α-KB per 1 mg protein per hour. Total protein content was obtained by the Bradford method (Bradford, 1976).

### 2.10 Determination of indole-3-acetic acid (IAA) production

IAA production was determined using a colorimetric assay based on the Salkowski reagent reaction. The reagent was prepared by adding 1 mL of 0.5 M FeCl_3_ to 49 mL of 35% H_2_SO4. Each bacterial isolate was inoculated into a nitrogen-free liquid medium supplemented with 0.01% L-tryptophan and incubated at 30 °C for 24 h with shaking at 150 rpm. After incubation, the cultures were centrifuged at 7,871 × g for 20 min. Subsequently, 70 μL of the supernatant was mixed with 140 μL of Salkowski reagent and incubated in the dark at room temperature for 20 min. The development of a pink color indicated the presence of IAA. The absorbance was measured at 530 nm using a spectrophotometer, and IAA concentration was determined using a standard calibration curve prepared with pure IAA. Nitrogen-free broth supplemented with L-tryptophan and Salkowski reagent was used as the control (Kumar et al., 2016).

### 2.11 Determination of gibberellic acid (GA3) production

Gibberellic acid (GA) production by the bacterial strains was determined following the method described by (Muromtsev and Nestyuk 1960). The strains were cultivated in liquid TS medium at 28 ± 2 °C on a shaker at 220 rpm. The amount of GA in the bacterial culture broth was measured for seven days. After incubation, the culture broth was filtered, and 1.0 mL of the filtrate was transferred into a 10 mL test tube. Then, 1.0 mL of Folin–Ciocalteu reagent was added and mixed thoroughly. The reaction mixture was incubated in the dark for 40 min at room temperature. The development of a light to dark green color indicated the presence of GA. The optical density of the samples was measured using a spectrophotometer at a wavelength of 750 nm. The GA concentration was determined based on the absorbance values as described by Woo et al. (2023).

### 2.12 Detection of protease activity

Protease activity was determined using a modified method described by Afrin and Bhuiyan (2023). The substrate solution consisted of hemoglobin prepared in citrate buffer (pH 5.5). Briefly, 0.5 mL of crude enzyme extract was mixed with 3 mL of hemoglobin solution and incubated at 37 °C for 30 min. The reaction was terminated by adding 3 mL of 5% trichloroacetic acid (TCA). The mixture was then filtered to remove precipitated proteins. Subsequently, ninhydrin reagent and 0.5 mL of distilled water were added to the filtrate and heated for 20 min. After cooling to room temperature, 5 mL of diluent solution (H_2_O:n-propanol, 1:1) was added. The absorbance of the resulting solution was measured at 570 nm using a spectrophotometer. Protease activity was estimated based on the amount of leucine released from hemoglobin. One unit of protease activity was defined as the amount of enzyme required to release 1 mg of leucine within 30 min at 37 °C. An enzyme extract–free reaction mixture was used as the control (Afrin and Bhuiyan, 2023).

### 2.13 Detection of chitinase activity

Chitinase activity was determined using the 3,5-dinitrosalicylic acid (DNS) assay with colloidal chitin as the substrate. Briefly, 1.0 mL of enzyme solution was mixed with 1.0 mL of 0.5% colloidal chitin prepared in 0.1 M citrate buffer (pH 7.0) and incubated at 37°C for 30 min in a shaking water bath. After incubation, 2 mL of DNS reagent was added to terminate the reaction, and the mixture was heated in a boiling water bath for 10 min. After cooling to room temperature, the reaction mixture was centrifuged at 9,800 × g for 10 minutes. The absorbance of the supernatant was measured at 540 nm using a spectrophotometer and compared with the control. One unit of chitinase activity was defined as the amount of enzyme required to release reducing sugars per minute per milliliter under the assay conditions (Xie et al., 2021).

### 2.14 Detection of amylase activity

The conventional DNSA (dinitrosalicylic acid) method was used for the quantitative analysis. Heating 1.4 g of 3,5-dinitrosalicylic acid in 70 mL of 0.5 N NaOH solution resulted in the formation of DNS reagent. After adding 30 g of sodium potassium tartrate (NaKC4O6·4H_2_O), the solution volume was raised to 1,000 mL. Then, the baseline solutions (maltose) and the experimental solutions (endophyte culture cell-free extract, 0.5 mL of 1% soluble starch solution, and 1 mL of DNS reagent) were added to the test tube and incubated for 15 min at 40 °C. 5 mL of distilled water was added, the mixture was cooled to room temperature, and the absorbance at 540 nm was determined and recorded. Based on these data, the amount of reducing sugars generated as a result of starch breakdown was calculated (Kim et al., 2025).

### 2.15 Detection of cellulase activity

Total cellulose enzyme activity was determined using a filter paper test. 6 cm × 1 cm pieces of Whatman Filter Paper No. 1 were cut, folded, and placed in labeled 2.0 mL microcentrifuge tubes. 500 μL of 50 mM pH 4.8 citrate buffer was added to the tubes, followed by 500 μL of enzyme (supernatant obtained in the Enzyme Isolation section). In control tubes, 500 μL of citrate buffer was added instead of enzyme. 1 g of glucose was dissolved in a 100 mL volumetric flask containing pH 4.8 citrate buffer. Serial dilution in 13 microcentrifuge tubes produced various glucose concentrations. The glucose concentrations ranged from 0 to 2.4 mg/mL. The tubes containing glucose standards and crude enzymes were incubated in a water bath at 50°C for 1 h, and the resultant reducing sugars were measured with DNS reagent (10 g 3,5-dinitrosalicylic acid, 0.5 g sodium sulfite, and 10 g NaOH). To stop the reaction, add 700 μL of DNS to each enzyme bioassay and standard tube. The tubes were then heated for 5 min and allowed to cool to room temperature. The enzymes, glucose standards, and negative control tubes received 300 μL of 400 g/L potassium sodium tartrate (PTS) solution. The absorbance of the resultant reducing sugars was measured at 540 nm using a SHIMADZU, UV1800 spectrophotometer (Gad et al., 2022).

### 2.16 Statistical analysis

Statistical analyses were performed using R statistical software. All experiments were conducted with four biological replicates (*n* = 5), and each measurement was performed in three technical replicates. Enzyme bioassay data were analyzed using two-way ANOVA followed by Tukey's HSD test to determine significant differences in enzyme production on the third, sixth, and 9th days of the experiment. Differences were considered statistically significant at *p* ≤ 0.05. The assumptions of normality and homogeneity of variance were considered prior to analysis.

## 3 Results

### 3.1 Isolation and characterization of endophytic bacteria

**Figure 1:**
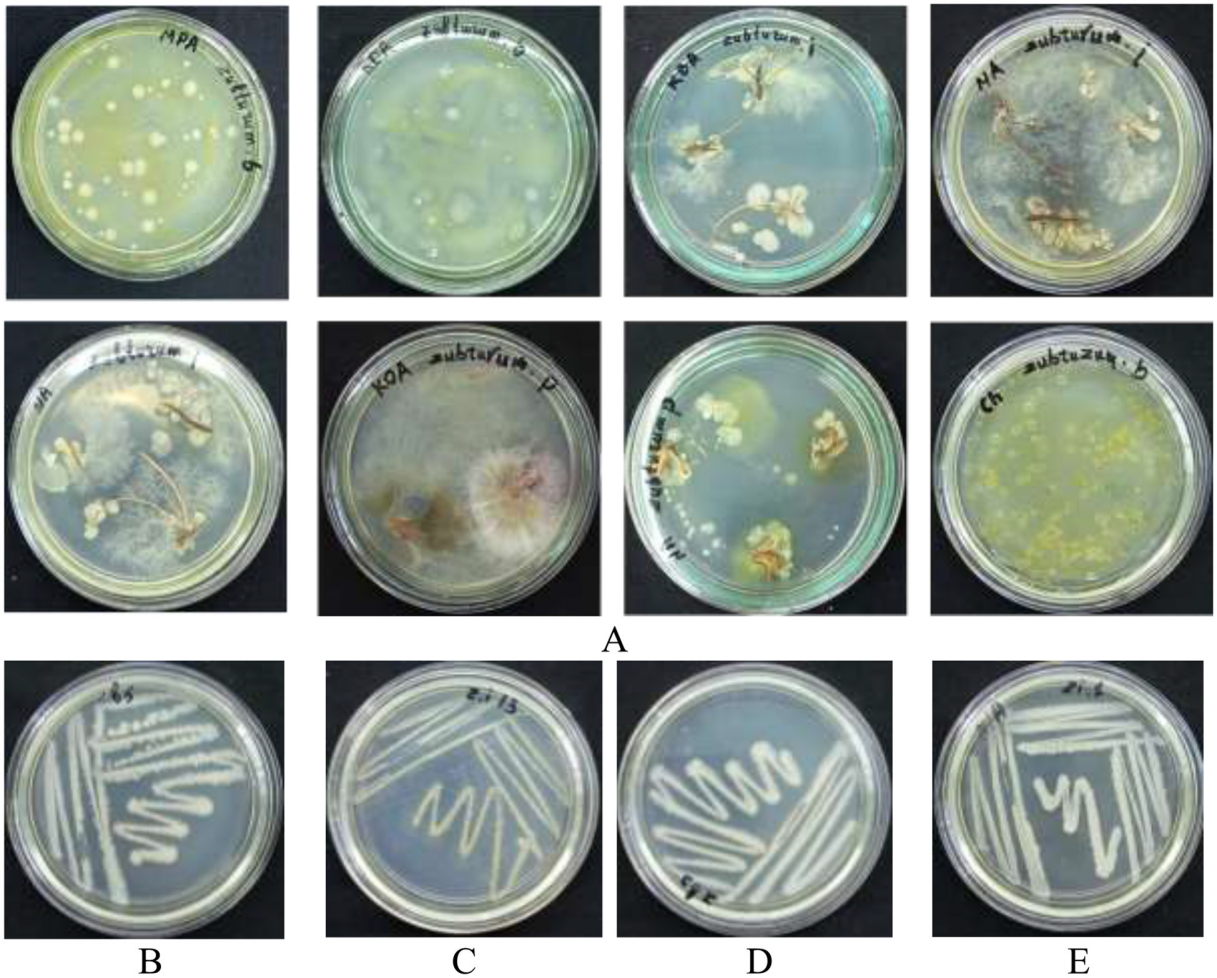
Representative images of endophytic bacterial isolation from roots, stems, and leaves of *Plantago lanceolata* L **(A)**. Surface-sterilized plant segments were placed on agar media (NA, PDA, MPA), resulting in the emergence of diverse bacterial colonies. Distinct colonies based on morphology were selected and purified through subculturing (**B**—leaf, **C**, **E**—root, **D**—stem).

Endophytic bacteria were isolated from the roots, stems, and leaves of *Plantago lanceolata* L. In total, 49 isolates were obtained, including 15 from roots, 10 from stems, and 24 from leaves (Figure 1). Isolates were coded according to their tissue of origin as ZI (root), ZP (stem), and ZB (leaf). Leaf tissues yielded the highest number of isolates. The results showed that the highest number of isolates was obtained from leaf tissues, indicating that this plant part provides relatively favorable conditions for the colonization and development of microorganisms. In contrast, the number of isolates recovered from roots and stems was moderate and low, respectively. The isolated bacteria were further evaluated for their morphological, biochemical, and plant growth-promoting properties. In addition, the isolates were screened for their ability to promote germination of *P. lanceolata* L. seeds in Petri dishes after 2 and 3 weeks.

### 3.2 Effect of endophytic bacteria on seed germination of *Plantago lanceolata*

**Figure 2:**
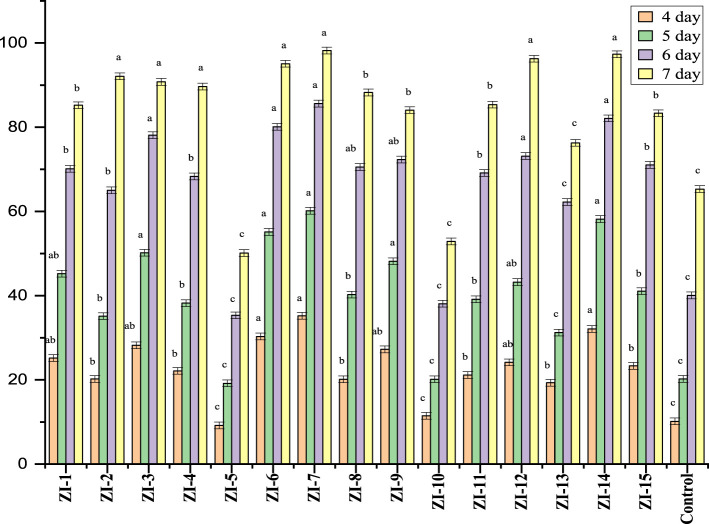
Effect of endophytic bacteria isolated from *P. lanceolata* L. on the seed germination of *P. lanceolata* L. Values represent mean ± SD (*n* = 5). Different letters (a, b, c) indicate significant differences between groups (*p* < 0.05, Duncan's multiple range test), while the same letter indicates no significant difference. Different letters (a, b, c) indicate significant differences between groups (*p* < 0.05, Duncan's multiple range test), while the same letter indicates no significant difference.

During the study, plantain seeds were treated with different isolates and the germination percentage of the seeds on days 4, 5, 6 and 7 as related to the control. The results showed that some bacterial isolates had a positive effect on seed germination, while others had a lower efficiency as related to the control. The maximum percentage of seed germination was observed on day 7 in isolates ZI-7 (98.21 ± 1.23 %), ZI-14 (97.33 ± 1.22 %), ZI-12 (96.27 ± 2.06 %) and ZI-6 (95.08 ± 2.41 %). These isolates were distinguished by their biological activity and significantly increased the germination rate and overall germination rate of the seeds. In particular, isolate ZI-7 achieved 35.22 ± 1.10 % germination on day 4, which is much higher than the control indicator of 10.15 ± 0.12 %. In addition, isolates ZI-3 (90.78 ± 1.15 %), ZI-2 (92.09 ± 1.03 %), ZI-8 (88.26 ± 2.17 %), ZI-4 (89.66 ± 1.96 %), and ZI-15 (83.33 ± 1.84 %) also showed high efficacy and had a positive effect compared to the control (65.31 ± 1.40 %). In general, at the end of day 7, most of the isolates were noted to significantly accelerate seed germination. But isolates ZI-5 and ZI-10 were found to have a negative effect on seed germination. These two isolates achieved only 50.11 ± 1.27 % and 52.88 ± 0.66 % seed germination, respectively, at day 7, which was the only sample lower than the control (65.31 ± 1.40 %) (Figure 2). Briefly, isolates such as ZI-7, ZI-14, and ZI-6 stand out as the most active bacteria, and it is suggested that they can be used as biological stimulants in the future.

**Figure 3:**
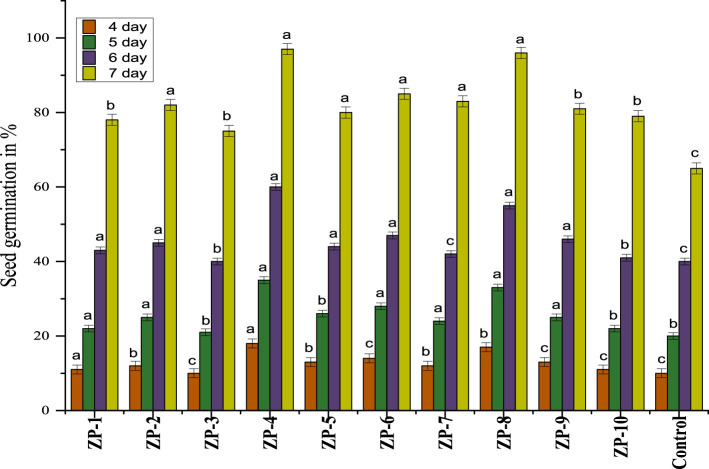
Effect of endophytic bacteria isolated from the stem of *P. lanceolata* L. on the seed germination of *P. lanceolata* L. Values represent mean ± SD (*n* = 5). Different letters (a, b, c) indicate significant differences between groups (*p* < 0.05, Duncan's multiple range test), while the same letter indicates no significant difference. Different letters (a, b, c) indicate significant differences between groups (*p* < 0.05, Duncan's multiple range test), while the same letter indicates no significant difference.

After treatment of plantain seeds with various bacterial isolates, the germination rates of the seeds were compared with the control (65.31 ± 1.40 %). The results of the study showed that all isolates had a certain positive effect on seed germination. Isolates ZP-4 and ZP-8 were the most active, reaching 97.22 ± 2.61 % and 96.67 ± 2.42 % germination percentages, respectively, at the end of the 7th day, which indicates their strong growth-promoting potential. The remaining isolates had values close to or slightly higher than the control and had a positive effect on seed germination in general. This confirms the possibility of using bacterial isolates as biologically active growth promoters (Figure 3).

**Figure 4:**
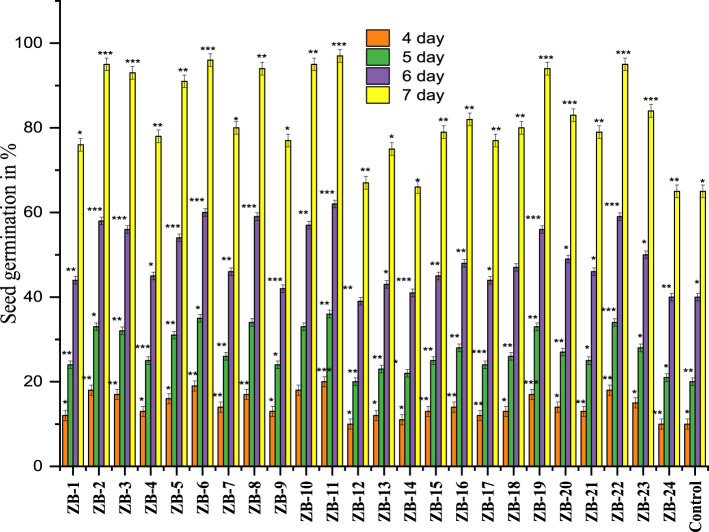
Effect of endophytic bacterial isolates from *Plantago lanceolata* L. leaves on seed germination percentage of *P. lanceolata* L. seeds after 7 days of incubation. Values represent mean ± SD (*n* = 5). Statistical significance was determined using ANOVA (*p* ≤ 0.05). *, **, and *** indicate statistically significant differences at *p* < 0.05, *p* < 0.01, and *p* < 0.001, respectively.

The results of the following study were aimed at studying the effect of 24 endophytic bacterial isolates isolated from the leaves of *P. lanceolata* L. on the germination of plantain seeds. The seeds were observed for 4 to 7 days, and the germination percentage was assessed. The control sample (without bacteria) achieved 10.97±0.13 % germination on day 4, 20.02±0.43 % on day 5, 39,97±0.53 % on day 6 and 65.66±2.38 % on day 7. The seeds treated with bacteria, on the other hand, showed higher germination rates than the control in most cases. In particular, isolates such as ZB-2, ZB-6, ZB-10, ZB-11, ZB-8, ZB-5, ZB-3, ZB-19 and ZB-22 significantly increased seed germination. These isolates showed germination rates of 91–97% at 7-day observation, which was 26.01±0.42 % to 32.75±0.77 % higher than the control sample. This indicates that they have high biological activity and growth-promoting properties. Also, isolates such as ZB-1, ZB-4, ZB-7, ZB-9, ZB-13, ZB-15, ZB-16, ZB-17, ZB-18, ZB-20, ZB-21, ZB-23 showed moderate activity, providing seed germination in the range of 75%−84% at the end of the 7th day. These isolates also had a positive effect compared to the control. In contrast, isolates ZB-12 (67.67±1.04 %), ZB-14 (66.34±1.37 %), and ZB-24 (65.12±2.09 %) had similar or equal seed germination to the control, and their effects were considered relatively low (Figure 4).

Overall, this study demonstrated that some endophytic bacteria significantly accelerated plantain seed germination, contributing to high percentage germination.

### 3.3 Quantitative production of indole-3-acetic acid (IAA) and gibberellic acid (GA)

According to the results of the study, the activity of gibberellic acid (GA) production of endophytic bacterial isolates gradually changed over time. In the 1st days, GA synthesis was recorded at a low level (0.33 ± 0.004–0.52 ± 0.01 mg mL^−1^), but on the 2 and 3 days it increased significantly, reaching 1.24 ± 0.02 mg mL^−1^ in some isolates. On the 4th day, maximum values were observed (ZI-7 −1.32 ± 0.01 mg mL^−1^, ZI-14 −1.15 ± 0.03 mg mL^−1^, ZB-3 −1.10 ± 0.02 mg mL^−1^, ZB-11 −0.98 ± 0.01 mg mL^−1^). On the 50th day, a decrease was observed in most isolates, which is explained by the transition of bacteria to the stationary phase or a decrease in nutrients. At the same time, all isolates produced 3–4 times more GA than the control treatment.

**Figure 5:**
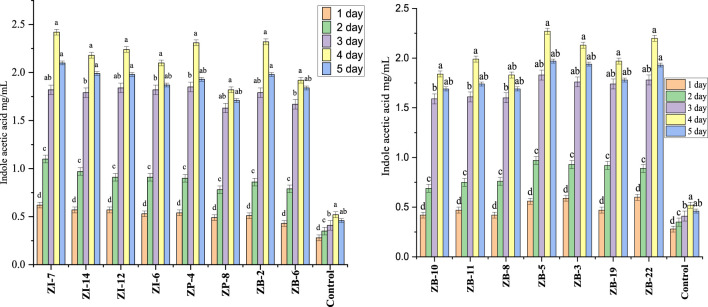
Quantitative production of indole-3-acetic acid (IAA) by endophytic bacterial isolates from *Plantago lanceolata* during 1–5 days of incubation. Hormone concentrations are expressed in mg mL^−1^. Values represent mean ± SD (n = 5). Different letters (a, b, c) indicate significant differences between groups (*p* < 0.05, Duncan's multiple range test), while the same letter indicates no significant difference. Different letters (a, b, c) indicate significant differences between groups (*p* < 0.05, Duncan's multiple range test), while the same letter indicates no significant difference.

**Figure 6:**
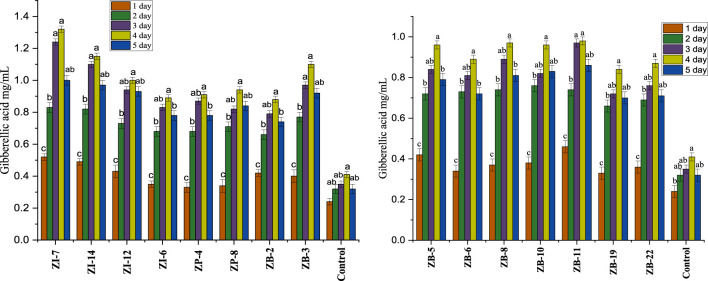
Quantitative production of gibberellic acid (GA) by endophytic bacterial isolates from *Plantago lanceolata* during 1–5 days of incubation. Hormone concentrations are expressed in mg mL^−1^. Values represent mean ± SD (*n* = 5). Different letters (a, b, c) indicate significant differences between groups (*p* < 0.05, Duncan's multiple range test), while the same letter indicates no significant difference. Different letters (a, b, c) indicate significant differences between groups (*p* < 0.05, Duncan's multiple range test), while the same letter indicates no significant difference.

The production of indole-3-acetic acid (IAA) by the endophytic bacterial isolates showed significant variation among the tested strains. In the control treatment, the IAA concentration was relatively low, ranging from 0.28 ± 0.003 to 0.52 ± 0.01 mg mL^−1^, whereas among the bacterial isolates it ranged from 0.42 ± 0.005–0.62 ± 0.01 mg mL^−1^ up to a maximum of 2.42 ± 0.03 mg mL^−1^. The highest IAA production was observed in isolates ZI-7 (2.42 ± 0.03 mg mL^−1^), ZB-2 (2.32 ± 0.03 mg mL^−1^), ZP-4 (2.31 ± 0.04 mg mL^−1^), and ZB-5 (2.27 ± 0.04 mg mL^−1^). Additionally, isolates ZI-14, ZI-12, ZB-3, and ZB-22 also demonstrated relatively high IAA production, ranging from 1.930.04 ± to 2.20 ± 0.06 mg/mL. Statistical analysis revealed that several isolates produced significantly higher levels of IAA compared with the control treatment (*p* < 0.05). Overall, these results indicate that some isolates, particularly ZI-7, ZI-14, ZB-3, ZB-11, ZB-2, and ZB-5, possess strong phytohormone-producing capacity (GA and IAA) and may serve as promising plant growth-promoting bioinoculants for stimulating plant growth (Figures 5, 6).

### 3.4 Antagonistic activity of endophytic bacteria against fungal pathogens

**Figure 7:**
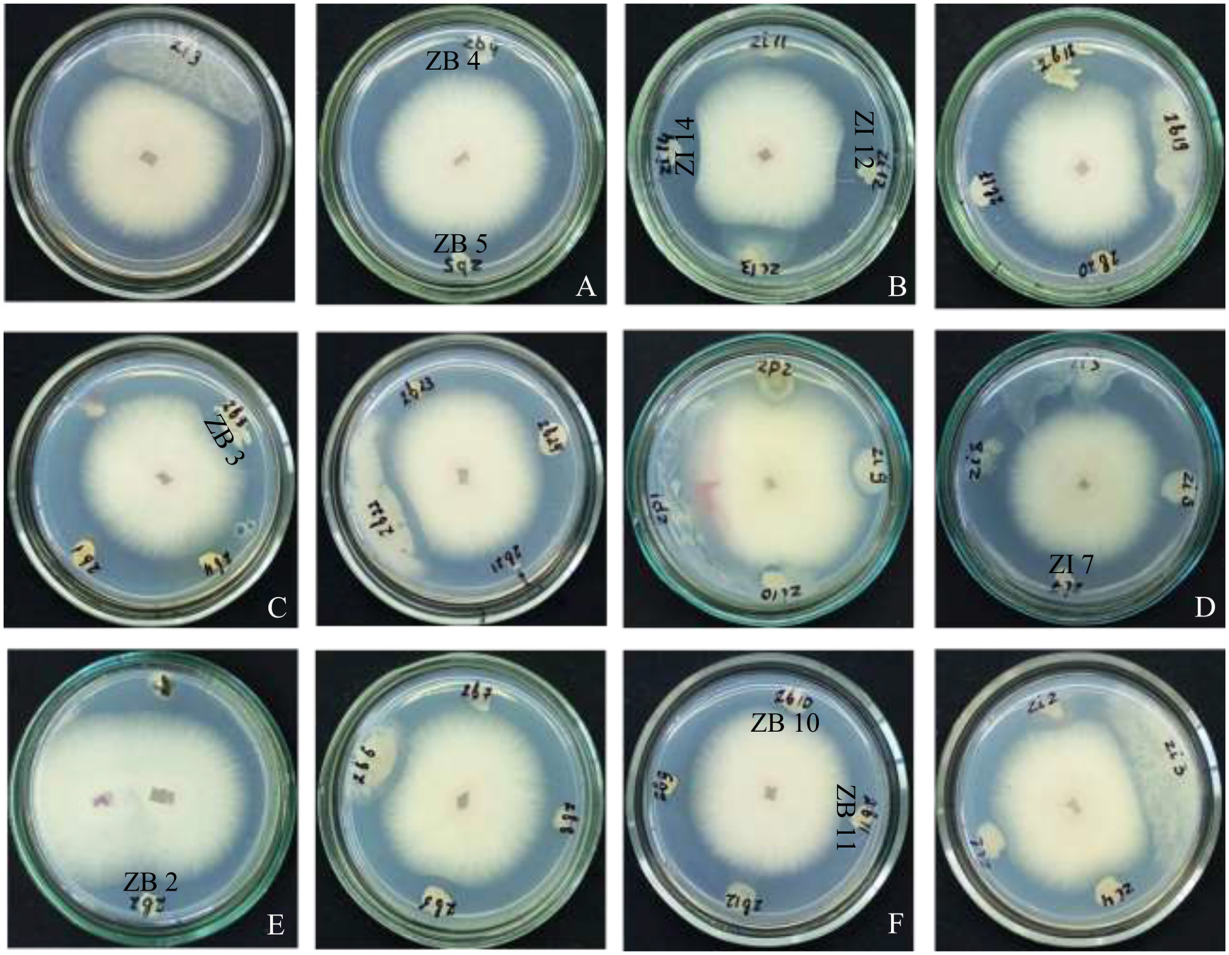
*In vitro* antagonistic activity of endophytic bacterial isolates against the phytopathogenic fungus *Fusarium solani* in a dual-culture assay. Clear inhibition zones around bacterial colonies indicate suppression of fungal growth. Representative plates showing the interaction between *F. solani* and bacterial isolates: ZB-4 **(A)**, ZB-5 **(A)**, ZI-12 **(B)**, ZI-14 **(B)**, ZB-3 **(C)**, ZI-7 **(D)**, ZB-2 **(E)**, ZB-11 **(F)**, and ZB-10 **(F)**.

The antagonistic activity of endophytic bacterial isolates against the phytopathogenic fungus *Fusarium solani* was evaluated *in vitro* using a dual-culture assay. The bacterial isolates were co-cultured with *F. solani* on Petri dishes, and the inhibition of fungal radial growth was measured as the diameter of the inhibition zone (mm) (Figure 7).

The results showed that several isolates exhibited strong antagonistic activity against *F. solani*. In particular, isolates ZI-12 (36.12 ± 0.87 mm) and ZI-14 (32.54 ± 0.76 mm) produced the largest inhibition zones, indicating strong suppression of fungal growth. Isolates ZI-7 and ZB-3 also demonstrated high antagonistic activity with inhibition zones of 28.76 ± 0.89 mm. In addition, isolates ZB-2, ZB-10, ZB-5, and ZB-11 exhibited moderate antifungal activity with inhibition zones of 25.11 ± 0.31, 24.15 ± 0.38, 24.67 ± 0.53, and 25.65 ± 0.63 mm, respectively. The antagonistic effect of isolate ZB-4 was comparatively lower (20.09 ± 0.43 mm).

**Figure 8:**
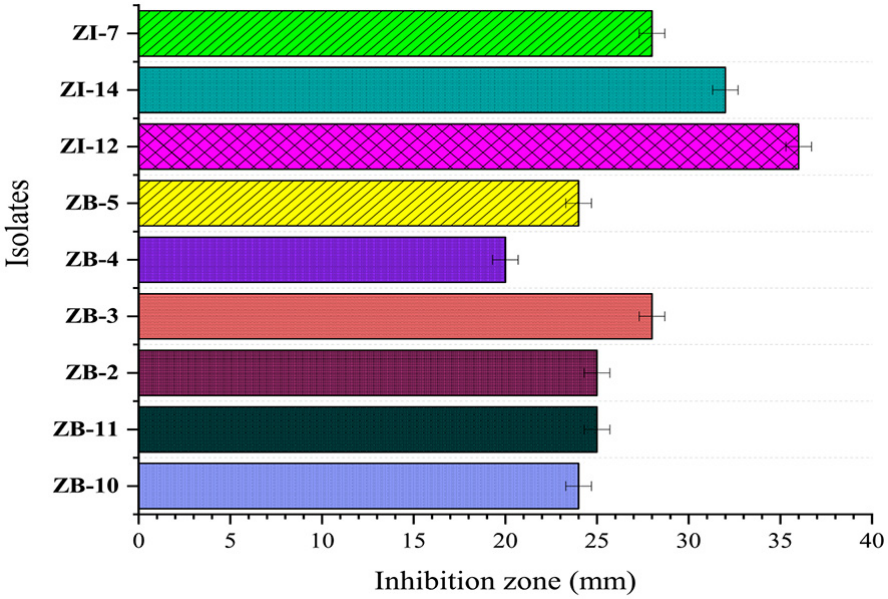
Antagonistic activity of selected endophytic bacterial isolates against the phytopathogenic fungus *Fusarium solani*. Antifungal activity was determined using a dual-culture assay and expressed as inhibition zone diameter (mm). Values represent mean ± standard deviation of three independent experiments. Different letters indicate significant differences among isolates according to one-way ANOVA followed by Tukey's test (*p* < 0.05). mean ± SD (*n* = 4).

Overall, these findings suggest that several endophytic bacterial isolates possess considerable antagonistic potential against *Fusarium solani*. In particular, isolates ZI-12 and ZI-14 demonstrated the strongest antifungal activity and may represent promising candidates for biological control of fungal pathogens (Figure 8).

As a result of the study, 9 out of 49 endophytic bacterial isolates exhibited strong antagonistic activity against *Fusarium solani*. These isolates effectively inhibited the growth of the pathogen *in vitro*, as indicated by the formation of clear inhibition zones around the bacterial colonies.

Among the nine active isolates, several strains belonging to the genera *Bacillus* and *Pseudomonas* were identified. Their antagonistic activity against *F. solani* indicates their potential for biological control applications. These findings confirm the antifungal activity of the isolates and highlight their possible use in environmentally friendly plant disease management. However, further greenhouse and field studies are required to confirm their practical applicability.

### 3.5 Molecular identification of endophytic bacterial isolates

A total of 49 bacterial isolates were obtained from three different tissues (roots, stems, and leaves) of *Plantago lanceolata* L. Based on preliminary screening for antifungal activity against phytopathogenic fungi and plant growth-promoting (PGP) traits, six endophytic bacterial isolates showing the highest biological activity were selected for further analysis.

The selected strains were identified based on 16S rRNA gene sequence analysis and compared with their closest relatives available in the GenBank database (NCBI). The identified endophytic strains belonged to two bacterial phyla: five isolates were assigned to the Firmicutes phylum, while one isolate belonged to the Proteobacteria phylum.

The Firmicutes isolates were representatives of the class *Bacilli*, including *Bacillus* sp. (ZI-14), *Bacillus* sp. (ZB-5), *Bacillus* sp. (ZI-3), *Brevibacillus* sp. (ZI-12), and *Brevibacillus* sp. (ZB-11). The Proteobacteria phylum was represented by *Pseudomonas* sp. (ZB-3), which belongs to the class Gammaproteobacteria. The identification results of the selected isolates are summarized in Table 1.

**Table 1:** Molecular identification of selected endophytic bacterial isolates from *Plantago lanceolata* L. based on 16S rRNA gene sequence analysis and comparison with reference sequences from the GenBank (NCBI) database.

Isolate code	Isolate code	Accession no. (Isolate)	Identified species	Closest NCBI match	Accession no. (NCBI)	Identity (%)
ZI14	IGPEB 4	PV848386.1	*Bacillus* sp.	*Bacillus cereus* strain *HBB532*	OM510462.1	98.95%
ZB5	IGPEB 5	PV848387.1	*Bacillus* sp.	*Bacillus pramycoides* strain *D12*	OQ510462.1	99.01%
ZI7	IGPEB 6	PV848388.1	*Bacillus* sp.	*Bacillus velezensis* strain *B13*	OQ941779.1	98.82%
ZI12	IGPEB 2	PV848384.1	*Brevibacillus* sp.	*Brevibacillus brevis* strain *44M*	MT783986.1	98.22%
ZB11	IGPEB 3	PV848385.1	*Brevibacillus* sp.	*Brevibacillus brevis* strain *D8.2*	JX286686.1	97.02%
ZB3	IGPEB 1	PV848383.1	*Pseudomonas* sp.	*Pseudomonas moraviensis* strain *WG406*	PQ781435.1	99.29%

Endophytic bacterial strains isolated in Uzbekistan were phylogenetically analyzed based on 16S rRNA gene sequences and compared with closely related sequences available in the NCBI database.

*Bacillus* sp. strain IGPEB 4 clustered with *Bacillus cereus* strain HBB532 and related strains, showing 98–99% sequence similarity. *Bacillus* sp. strain IGPEB 5 was closely related to *Bacillus pramycoides* strain D12. *Bacillus* sp. strain IGPEB 6 grouped with *Bacillus velezensis* strain B13 and related isolates, indicating high genetic similarity.

**Figure 9:**
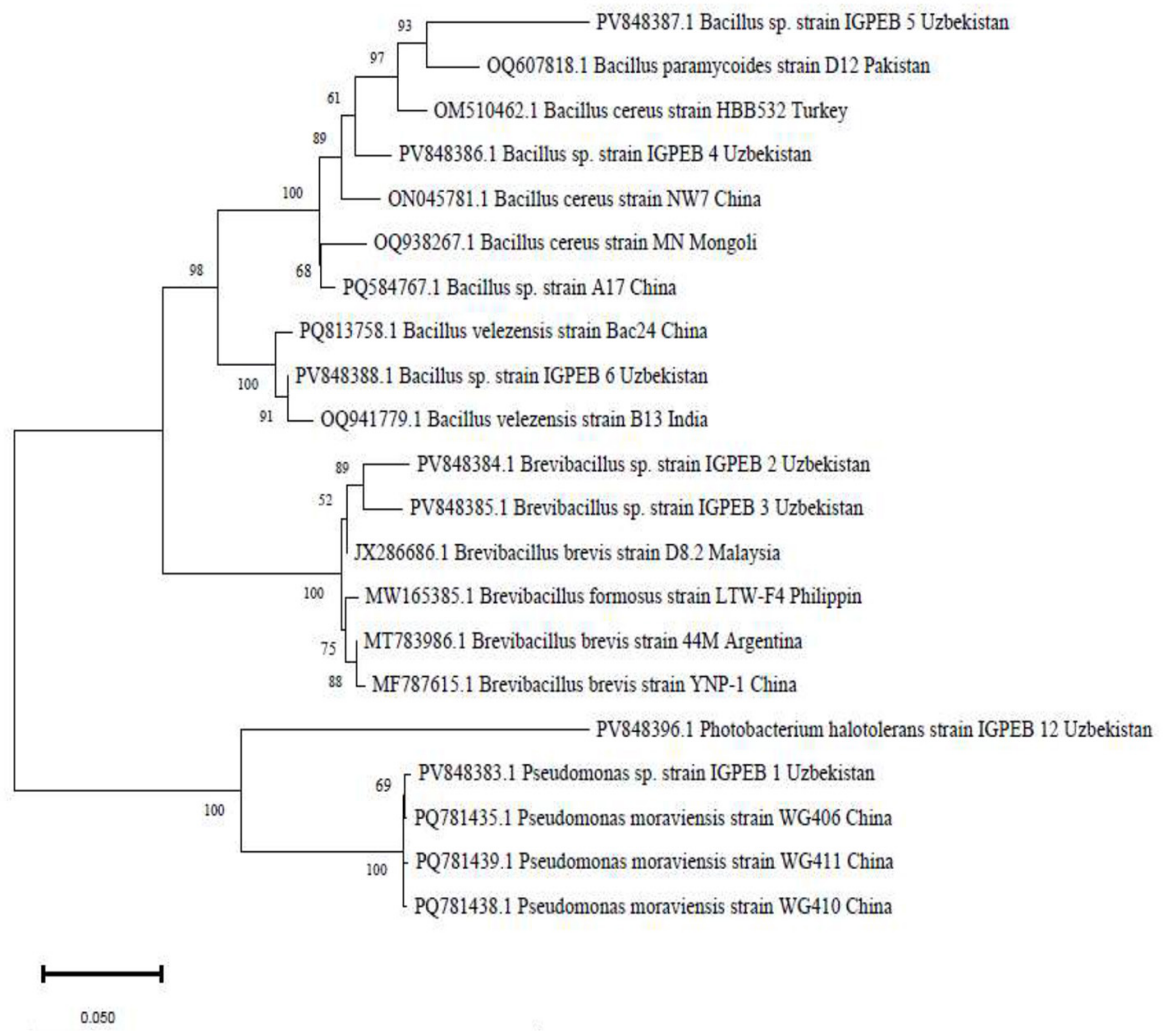
Phylogenetic dendrogram based on partial 16S rRNA gene sequences showing the relationships between the endophytic bacterial isolates obtained from *Plantago lanceolata* L. and closely related reference strains retrieved from the NCBI GenBank database. The tree was constructed using the Neighbor-Joining method, and bootstrap values (%) based on 1,000 replicates are shown at the branch nodes.

*Brevibacillus* sp. strains IGPEB 2 and IGPEB 3 clustered with *Brevibacillus brevis* strains reported from different geographical regions. *Pseudomonas* sp. strain IGPEB 1 showed close phylogenetic affinity to *Pseudomonas moraviensis* strains (Figure 9).

### 3.6 Phosphate (P) and zinc (Zn) solubilization by endophytic bacteria

The results of the study showed that *Bacillus* sp. strain IGPEB6 exhibited the highest phosphate solubilization index among the tested isolates. *Bacillus* sp. strain IGPEB4 and *Bacillus* sp. strain IGPEB5 demonstrated moderate phosphate solubilization activity. In contrast, *Brevibacillus* sp. strain IGPEB2 and *Brevibacillus* sp. strain IGPEB3 showed relatively low phosphate solubilization ability, while *Pseudomonas* sp. strain IGPEB1 recorded the lowest index (Table 2).

**Table 2:** Zinc (Zn) and phosphate (P) solubilization indices (SI), hydrogen cyanide (HCN) production, and siderophore production of selected endophytic bacterial isolates obtained from *Plantago lanceolata* L. mean ± SD (*n* = 5).

Isolates	Zinc carbonate SI (mm)	Zinc oxide SI (mm)	Zinc phosphate SI (mm)	Phosphate SI (mm)	HCN qualitative	HCN quantitative (OD)	Siderophore Percent (psu)
*Bacillus* sp. strain IGPEB 4	4.4 ± 0.059	3.74 ± 0.072	2.2 ± 0.035	2.9 ± 0.059	+++	0.041 ± 0.002	64.28 ± 0.72
*Bacillus* sp. strain IGPEB 5	3.1 ± 0.065	1.7 ± 0.040	1.2 ± 0.027	1.8 ± 0.021	++	0.034 ± 0.002	41.13 ± 0.57
*Bacillus* sp. strain IGPEB 6	5.1 ± 0.058	6.1 ± 0.084	2.2 ± 0.034	3.4 ± 0.057	++++	0.073 ± 0.003	78.12 ± 0.68
*Brevibacillus* sp. strain IGPEB 2	2.9 ± 0.034	2.8 ± 0.095	1.7 ± 0.032	1.9 ± 0.051	++	0.024 ± 0.002	72.81 ± 0.61
*Brevibacillus* sp. strain IGPEB 3	1.7 ± 0.040	2.5 ± 0.061	1.4 ± 0.033	2.0 ± 0.042	++	0.033 ± 0.002	47.45 ± 0.66
*Pseudomonas* sp. strain IGPEB 1	2.5 ± 0.052	1.9 ± 0.068	1.8 ± 0.032	1.7 ± 0.035	+	0.013 ± 0.003	22.6 ± 0.53

In the zinc solubilization assay, isolates belonging to the genera *Bacillus* and *Brevibacillus* showed relatively higher solubilization activity. Among them, *Bacillus* sp. strain IGPEB6 exhibited the strongest zinc solubilization ability. In contrast, *Pseudomonas* sp. strain IGPEB1 showed the lowest zinc solubilization activity.

Zinc solubilization differed depending on the zinc compound. The highest zinc oxide solubilization was observed in *Bacillus* sp. IGPEB 6 (6.10 ± 0.08 mm), while *Bacillus* sp. IGPEB 4 showed strong activity for zinc carbonate (4.40 ± 0.06 mm). Zinc phosphate solubilization was comparatively lower across all isolates, with maximum activity recorded in *Bacillus* sp. IGPEB 4 and IGPEB 6 (2.20 ± 0.04 mm).

Phosphate solubilization ranged from 1.70 ± 0.04 mm to 3.40 ± 0.06 mm, with the highest activity observed in *Bacillus* sp. IGPEB 6, followed by *Bacillus* sp. IGPEB 4.

All isolates were capable of producing HCN, although at varying levels. The strongest qualitative HCN production (++++) and the highest quantitative value (0.073 ± 0.003 OD) were recorded in *Bacillus* sp. IGPEB 6, whereas *Pseudomonas* sp. IGPEB 1 showed the lowest production (+; 0.013 ± 0.003 OD).

### 3.7 Siderophore and hydrogen cyanide (HCN) production by endophytic bacteria

Siderophore production was detected in all isolates, ranging from 22.60 ± 0.53% to 78.12 ± 0.68%. The highest production was observed in *Bacillus* sp. IGPEB 6, followed by *Brevibacillus* sp. IGPEB 2 and *Bacillus* sp. IGPEB 4, while *Pseudomonas* sp. IGPEB 1 exhibited the lowest level.

The hydrogen cyanide (HCN) production capacity of the bacterial endophytes was assessed using both qualitative and quantitative analyses. *Bacillus* sp. strain IGPEB4 showed strong (+++) HCN production with a quantitative value of 0.041 ± 0.002 OD, while *Bacillus* sp. strain IGPEB5 showed moderate (++) production with a value of 0.034 ± 0.002 OD. The highest HCN production was observed in *Bacillus* sp. strain IGPEB6, which showed very strong (++++) qualitative production with an OD value of 0.073 ± 0.003.

*Brevibacillus* sp. strain IGPEB2 (0.024 ± 0.002 OD) and *Brevibacillus* sp. strain IGPEB3 (0.033 ± 0.002 OD) also showed moderate (++) HCN production. In contrast, *Pseudomonas* sp. strain IGPEB1 exhibited weak (+) HCN production with a quantitative value of 0.013 ± 0.003 OD (Table 2).

### 3.8 Total cellulase (FPase) activity of endophytic bacteria

The production of total cellulase (FPase) by the endophytic bacterial isolates varied significantly over time (*p* < 0.05). In general, most isolates showed an increase in enzyme activity from day 2 to day 6, followed by a decrease on day 7. Each isolate exhibited distinct dynamics in cellulase production, with maximum activity observed at different time points. In this study, the classification of isolates as high, moderate, or low cellulase producers was based on the relative comparison of FPase activity values obtained for each isolate.

Among the tested isolates, *Bacillus* sp. strain IGPEB 6 demonstrated the highest cellulase production potential. Enzyme activity increased from 5.63 ± 0.07 IU mL^−1^ on day 2 to 21.69 ± 0.54 IU mL^−1^ on day 5, reaching a maximum value of 25.91 ± 0.63 IU mL^−1^ on day 6 before decreasing to 16.89 ± 0.28 IU mL^−1^ on day 7. Similarly, *Bacillus* sp. strain IGPEB 4 showed a significant increase in cellulase activity from 4.35 ± 0.09 IU mL^−1^ on day 2 to 19.33 ± 0.24 IU mL^−1^ on day 5, with peak activity of 21.25 ± 0.42 IU mL^−1^ on day 6, followed by a decrease to 14.30 ± 0.35 IU mL^−1^ on day 7. Based on these results, these isolates were considered high cellulase producers.

Moderate enzyme production was observed in *Brevibacillus* sp. strain IGPEB 2 and *Brevibacillus* sp. strain IGPEB 3. In *Brevibacillus* sp. strain IGPEB 2, cellulase activity increased from 3.71 ± 0.08 IU mL^−1^ on day 2 to 14.52 ± 0.32 IU mL^−1^ on day 5, followed by a gradual decline to 12.65±0.27 IU mL^−1^ and 9.52 ± 0.28 IU mL^−1^ on days 6 and 7, respectively. Similarly, *Brevibacillus* sp. strain IGPEB 3 showed an increase from 2.38 ± 0.05 IU mL^−1^ on day 2 to a maximum of 13.87 ± 0.28 IU/mL on day 6 before decreasing to 8.43 ± 0.21 IU mL^−1^ on day 7. These isolates were categorized as moderate cellulase producers based on their intermediate enzyme activity.

Lower cellulase activity was observed in *Pseudomonas* sp. strain IGPEB 1, which increased from 1.28 ± 0.03 IU mL^−1^ on day 2 to a maximum of 10.93 ± 0.23 IU mL^−1^ on day 6 and then decreased to 7.92 ± 0.25 IU mL^−1^ on day 7. Due to the comparatively lower FPase activity values, this isolate was classified as a low cellulase producer. Overall, the results indicate that *Bacillus* sp. strain IGPEB 6 exhibited significantly higher cellulase activity compared to the other isolates, reaching the highest value of 25.9 ± 0.33 IU mL^−1^ on day 6 (Table 3).

**Table 3:** Total cellulase (FPase) activity produced by endophytic bacterial isolates during different incubation days.

Strain	Day 2 enzyme units (IU/mL)	Day 3 enzyme units (IU/mL)	Day 4 enzyme units (IU/mL)	Day 5 enzyme units (IU/mL)	Day 6 enzyme units (IU/mL)	Day 7 enzyme units (IU/mL)
*Bacillus* sp. strain IGPEB 4	4.35 ± 0.09^de^	9.06 ± 0.27^de^	13.25 ± 0.16^c^	19.33 ± 0.24^de^	21.25 ± 0.42^c^	14.30 ± 0.35^c^
*Bacillus* sp. strain IGPEB 5	2.18 ± 0.08^a^	7.21 ± 0.22^a^	11.79 ± 0.28^a^	17.9 ± 0.33^a^	15.20 ± 0.38^a^	10.3 ± 0.23^a^
*Bacillus* sp. strain IGPEB 6	5.63 ± 0.07^b^	10.87 ± 0.23^b^	15.26 ± 0.34^b^	21.69 ± 0.54^b^	25.91 ± 0.63^b^	16.89 ± 0.28^b^
*Brevibacillus* sp. strain IGPEB 2	3.71 ± 0.08^de^	3.17 ± 0.12^de^	10.54 ± 0.25^e^	14.52 ± 0.32^e^	12.65 ± 0.2^de^	9.52 ± 0.28^e^
*Brevibacillus* sp. strain IGPEB 3	2.38 ± 0.05^de^	6.27 ± 0.20^de^	10.73 ± 0.26^d^	11.57 ± 0.42^de^	13.87 ± 0.28^d^	8.43 ± 0.21^d^
*Pseudomonas* sp. strain IGPEB 1	1.28 ± 0.03^c^	5.42 ± 0.18^c^	8.13 ± 0.19^c^	9.68 ± 0.48^c^	10.93 ± 0.23^c^	7.92 ± 0.25^c^

Values are expressed as mean ± standard deviation of three independent experiments. Different letters indicate statistically significant differences between treatments (*p* < 0.05). mean ± SD (*n* = 5).

### 3.9 ACC deaminase activity of endophytic bacteria

Almost all six endophytic bacterial isolates exhibited ACC deaminase activity when ACC was added to DF minimal salt broth medium as the sole nitrogen source. The enzyme activity ranged from 9.72 ± 0.12 to 16.26 ± 0.34 μmol α-ketobutyrate mg protein^−1^ h^−1^.

Among the tested isolates, *Bacillus* sp. strain IGPEB 6 and *Pseudomonas* sp. strain IGPEB 1 showed the highest ACC deaminase activity, with values of 15.95±0.58 and 16.26±0.34 μmol α-ketobutyrate mg protein^−1^ h^−1^, respectively. Intermediate activity levels were observed in *Bacillus* sp. strain IGPEB 5 (14.31 ± 0.18 μmol α-ketobutyrate mg protein^−1^ h^−1^) and *Brevibacillus* sp. strain IGPEB 2 (14.89 ± 0.32 μmol α-ketobutyrate mg protein^−1^ h^−1^). Lower enzyme activity was detected in *Bacillus* sp. strain IGPEB 4 (12.62 ± 0.15 μmol α-ketobutyrate mg protein^−1^ h^−1^) and *Brevibacillus* sp. strain IGPEB 3 (9.72 ± 0.24 μmol α-ketobutyrate mg protein^−1^ h^−1^).

**Figure 10:**
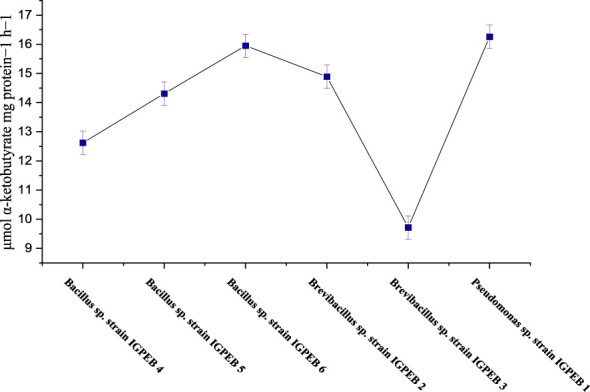
ACC deaminase activity of endophytic bacterial isolates. Enzyme activity was determined based on α-ketobutyrate production and expressed as μmol α-ketobutyrate mg protein^−1^ h^−1^. Values represent the mean ± standard deviation of three independent experiments. Error bars indicate standard deviation. mean ± SD (*n* = 5).

In the absence of ACC, only two isolates retained the ability to produce ACC deaminase, with α-ketobutyrate concentrations ranging from 0.012 ± 0.0001 to 0.074 ± 0.0011 μmol α-ketobutyrate mg protein^−1^ h^−1^. These results indicate that the presence of ACC as the sole nitrogen source significantly stimulates ACC deaminase biosynthesis in the tested endophytic bacterial isolates, which may contribute to enhanced stress tolerance in plants (Figure 10).

### 3.10 Enzymatic activities of endophytic bacteria

The selected bacterial isolates were evaluated for their chitinase, protease, and amylase activities. The results showed that all isolates were capable of producing the three enzymes, although their activity levels varied among the strains.

The highest chitinase activity was observed in *Bacillus* sp. strain IGPEB 6 (4.12 ± 0.22 U mL^−1^), which was significantly higher than the activity recorded for the other isolates. *Bacillus* sp. strain IGPEB 4 also showed relatively high chitinase activity (3.97 ± 0.16 U mL^−1^). Moderate activity was detected in *Pseudomonas* sp. strain IGPEB 1 (3.10 ± 0.20 U mL^−1^), whereas *Brevibacillus* sp. strain IGPEB 2 and *Brevibacillus* sp. strain IGPEB 3 showed comparatively lower chitinase activities (2.94–2.96 U mL^−1^) (Table 4).

**Table 4:** *Enzymatic activity of selected endophytic bacteria* mean ± SD (*n* = 5).

Strain	Chitinase U mL^−1^	Protease U mL^−1^	Amylase U mL^−1^
*Bacillus* sp. strain IGPEB 4	3.97 ± 0.16	2.34 ± 0.08	4.17 ± 0.1
*Bacillus* sp. strain IGPEB 5	2.89 ± 0.14	2.2 ± 0.09	4.1 ± 0.1
*Bacillus* sp. strain IGPEB 6	4.12 ± 0.22	2.42 ± 0.13	4.23 ± 0.12
*Brevibacillus* sp. strain IGPEB 2	2.94 ± 0.13	1.93 ± 0.07	3.86 ± 0.09
*Brevibacillus* sp. strain IGPEB 3	2.96 ± 0.34	1.92 ± 0.09	3.98 ± 0.01
*Pseudomonas* sp. strain IGPEB 1	3.1 ± 0.20	2.26 ± 0.09	3.91 ± 0.01

Protease production also differed among the isolates. The highest protease activity was recorded in *Bacillus* sp. strain IGPEB 6 (2.42 ± 0.13 U mL^−1^), followed by *Bacillus* sp. strain IGPEB 4 (2.34 ± 0.08 U mL^−1^) and *Pseudomonas* sp. strain IGPEB 1 (2.26 ± 0.09 U mL^−1^). *Bacillus* sp. strain IGPEB 5 exhibited a protease activity of 2.20 ± 0.09 U mL^−1^, which was comparable to the activity observed in *Pseudomonas* sp. strain IGPEB 1. The lowest values were recorded for *Brevibacillus* sp. strain IGPEB 2 and *Brevibacillus* sp. strain IGPEB 3 (1.92–1.93 U mL^−1^) (Table 4).

A comparable trend was observed for amylase production. The highest activities were detected in *Bacillus* sp. strain IGPEB 6 (4.23 ± 0.12 U mL^−1^) and *Bacillus* sp. strain IGPEB 4 (4.17 ± 0.10 U mL^−1^). *Bacillus* sp. strain IGPEB 5 also showed relatively high amylase activity (4.10 ± 0.10 U mL^−1^), which was close to the values observed for the other *Bacillus* isolates. In contrast, *Pseudomonas* sp. strain IGPEB 1 (3.91 ± 0.01 U mL^−1^), *Brevibacillus* sp. strain IGPEB 2 (3.86 ± 0.09 U mL^−1^), and *Brevibacillus* sp. strain IGPEB 3 (3.98 ± 0.01 U mL^−1^) exhibited comparatively lower amylase activities (Table 4).

Overall, *Bacillus* sp. strain IGPEB 6 showed the highest enzymatic activity for all three enzymes, indicating its strong enzymatic potential. *Bacillus* sp. strain IGPEB 4 also demonstrated high chitinase and protease activities. In contrast, isolates belonging to the genus *Brevibacillus* exhibited relatively lower activity for the tested enzymes.

## 4 Discussion

This study provides new scientific results in the extreme agroecological conditions of the understudied Surkhandarya region by combining organ-specific analysis of endophytic bacteria associated with *Plantago lanceolata* L. with a comprehensive assessment of plant growth-promoting properties. The results revealed an endophytic community primarily composed of members of the phyla Firmicutes (*Bacillus* and *Brevibacillus*) and Proteobacteria (*Pseudomonas*). These isolates demonstrated multiple PGP traits, including phytohormone production, nutrient solubilization, antifungal activity, and the production of various hydrolytic enzymes. The successful isolation and molecular identification of these strains, together with the assignment of GenBank accession numbers, expands our understanding of the microbiome associated with *P. lanceolata* L. and highlights their potential for the development of sustainable bioinoculants.

A total of 49 endophytic bacterial strains were isolated from the roots, stems, and leaves of *P. lanceolata* L. The majority of isolates were obtained from leaf tissues (24), followed by roots (15) and stems (10). This distribution pattern is consistent with previous studies reporting distinct microbial communities in different plant tissues due to tissue-specific selective pressures (Rani et al., 2022). The unique physicochemical conditions of the phyllosphere and the constant exposure of leaves to environmental microorganisms may contribute to the greater diversity observed in aerial tissues.

Six representative isolates (*Pseudomonas* sp. strain IGPEB1, *Brevibacillus* sp. strain IGPEB2, *Brevibacillus* sp. strain IGPEB3, *Bacillus* sp. strain IGPEB4, *Bacillus* sp. strain IGPEB5, and *Bacillus* sp. strain IGPEB6) were further identified through molecular characterization using 16S rRNA gene sequence analysis. Phylogenetic analysis revealed that the isolates were closely related to members of the genera *Pseudomonas, Brevibacillus*, and *Bacillus*. Similar findings have been reported in studies investigating endophytic and rhizospheric microbial communities associated with medicinal plants, where members of the genera *Bacillus* and *Pseudomonas* frequently dominate (Krimi et al., 2016; Jabborova et al., 2021a,b; Duhan et al., 2020; Jabborova et al., 2022; Mamarasulov et al., 2022; Wang et al., 2023; Sharma, 2024; Jabborova et al., 2025b). These bacterial taxa are well known for their metabolic versatility and resilience, particularly due to the spore-forming ability of *Bacillus* and the metabolic diversity of *Pseudomonas*, which contribute to their strong PGP potential (de Lima et al., 2024; Frans et al., 2025). The phylogenetic relationships between our isolates and strains reported from various geographical regions, including China, India, and Pakistan, further indicate the widespread distribution and ecological adaptability of these bacterial taxa.

One of the key findings of this study is the ability of the isolated endophytes to produce phytohormones such as indole-3-acetic acid (IAA) and gibberellic acid (GA). These phytohormones play a critical role in plant growth regulation. IAA promotes root elongation and branching, thereby improving nutrient and water uptake (Gupta et al., 2022; Shreshtha et al., 2024), while gibberellins are involved in stem elongation, seed germination, and the regulation of developmental processes (Woo et al., 2023). The high phytohormone production observed in several isolates, particularly *Bacillus* sp. strain IGPEB6 and *Bacillus* sp. strain IGPEB4, was closely associated with enhanced seed germination of *P. lanceolata* L., reaching up to 98% compared with 65% in the control. These findings are consistent with previous studies demonstrating that IAA-producing endophytic bacteria can significantly enhance seed germination and early seedling growth (Vendan et al., 2010; Pal et al., 2024).

In addition to phytohormone production, the isolates demonstrated a strong ability to improve plant nutrient availability through phosphate and zinc solubilization (Li et al., 2025). In many soils, these nutrients occur in insoluble forms that are not readily available to plants. The ability of bacterial isolates to solubilize these minerals is therefore essential for improving plant nutrient acquisition. Phosphate solubilization is typically mediated by the secretion of organic acids such as gluconic and citric acids, which lower the pH of the surrounding environment and release soluble orthophosphate (Gupta et al., 2024). Similarly, zinc-solubilizing bacteria contribute to alleviating micronutrient deficiencies commonly observed in agricultural soils.

The antagonistic activity of several isolates against *Fusarium solani* further highlights their potential as biological control agents. Significant inhibition zones were observed *in vitro*, indicating strong antifungal activity. This antagonistic effect may be attributed to multiple mechanisms, including the production of hydrolytic enzymes such as chitinases, proteases, and cellulases, which degrade fungal cell walls (Riseh et al., 2024; Ramírez et al., 2024; Gayathri et al., 2025; Baltruschat et al., 2025). In addition, siderophore production may enhance the competitive ability of these bacteria by sequestering iron from the surrounding environment, thereby limiting its availability to pathogenic fungi (Sarvepalli et al., 2023). Similar biocontrol activity of endophytic *Bacillus* and *Pseudomonas* isolates associated with *P. lanceolata* L. has been previously reported (Krimi et al., 2016; Rosini et al., 2025). These characteristics suggest that certain isolates, particularly *Bacillus* sp. strain IGPEB6 and *Bacillus* sp. strain IGPEB4, may serve as promising candidates for the development of environmentally friendly biocontrol agents aimed at managing soil-borne pathogens and reducing the use of chemical pesticides (Sherzad et al., 2025; Vimal et al., 2025).

The enzymatic profile of the isolates further indicates their potential role in nutrient cycling and stress mitigation. The production of extracellular enzymes such as cellulase, amylase, chitinase, and protease facilitates the degradation of complex organic substrates, thereby contributing to improved soil fertility and nutrient availability. In particular, high cellulase activity observed in *Bacillus* sp. strain IGPEB6 suggests its potential application in biomass degradation processes (Gad et al., 2022).

Another important trait identified in this study was ACC deaminase activity. This enzyme degrades the plant ethylene precursor 1-aminocyclopropane-1-carboxylate (ACC), thereby reducing stress-induced ethylene levels in plants (Penrose and Glick, 2003). Endophytic bacteria that produce ACC deaminase support plant growth under stress conditions by regulating ethylene levels. This enzyme reduces ethylene concentrations, thereby mitigating the effects of drought, salinity, and heavy metal stress and helping to maintain plant growth (Shahid et al., 2023). It has also been reported that such bacteria positively influence root development and increase total biomass (Grobelak et al., 2018).

Overall, the findings of this study demonstrate that endophytic bacteria associated with *P. lanceolata* L. possess multiple plant growth-promoting traits that may contribute to plant development, nutrient acquisition, and stress tolerance. These multifunctional characteristics highlight their potential for application in sustainable agriculture, particularly in the development of microbial bioformulations aimed at enhancing crop productivity while minimizing environmental impacts (Finkel et al., 2017; Santoyo et al., 2021).

Recent recommendations indicate that studies investigating plant growth-promoting bacteria should integrate *in vitro* screening with subsequent validation under greenhouse and field conditions to ensure reliable interpretation of their agricultural potential (de-Bashan and Nannipieri, 2024). In the present study, the identified endophytic isolates associated with *Plantago lanceolata* L. exhibited several plant growth-promoting traits under laboratory conditions. However, further greenhouse and field experiments will be necessary to confirm their effectiveness and practical applicability under natural environmental conditions.

## 5 Conclusion

This study provides insight into the diversity and functional potential of endophytic bacteria associated with *Plantago lanceolata* L. under the conditions studied. The isolated strains, in particular *Bacillus* sp. IGPEB6 and *Bacillus* sp. IGPEB4, exhibited several plant growth-promoting properties, such as phytohormone production, nutrient solubilization, antifungal activity, and hydrolytic enzyme production. These findings suggest that endophytic isolates may contribute to plant growth through direct mechanisms such as phytohormone production and nutrient mobilization, and indirect mechanisms such as pathogen suppression and stress alleviation. The presence of multiple plant growth-promoting properties suggests the potential for integrated functional roles in supporting plant development. However, given the limited environmental characterization and sample size, the results should be interpreted with caution and limited to the conditions studied. Further studies are needed to confirm these findings under different environmental conditions. The observed properties highlight the potential of these bacterial strains as candidates for the development of environmentally friendly biofertilizers and biocontrol agents. However, their efficacy needs to be confirmed through experiments conducted under greenhouse and field conditions. Future studies could focus on the development of sustainable bioformulations and the evaluation of their efficacy across different crops, including *P. lanceolata* L. In addition, more robust taxonomic approaches, such as multilocus sequence analysis (MLSA), *gyrB* or *rpoB* gene sequencing, or whole-genome sequencing, should be incorporated to achieve more accurate species-level identification of the isolates.

## Data Availability

The original contributions presented in the study are included in the article/supplementary material, further inquiries can be directed to the corresponding author/s.

## References

[B1] Abbas M. M. Ismael W. H. Mahfouz A. Y. Daigham G. E. Attia M. S. (2024). Efficacy of endophytic bacteria as promising inducers for enhancing the immune responses in tomato plants and managing Rhizoctonia root-rot disease. Sci. Rep. 14:1331. doi: 10.1038/s41598-023-51000-8 38225343 PMC10789748

[B2] Abd El-Rahman A. F. Shaheen H. A. Abd El-Aziz R. M. Ibrahim D. S. (2019). Influence of hydrogen cyanide-producing rhizobacteria in controlling the crown gall and root-knot nematode, *Meloidogyne incognita*. Egypt. J. Biol. Pest Control 29:41. doi: 10.1186/s41938-019-0143-7

[B3] Abdusamatov S. Jabborova D. Azimova N. Alimov J. E. Djalolova B. Alhewairini S. S. . (2025a). Eco-friendly biodegradation of plant biomass using multienzyme-producing trichoderma harzianum for sustainable feed improvement. Waste Biomass Valor. 17, 1835. doi: 10.1007/s12649-025-03132-3

[B4] Abdusamatov S. Jabborova D. Mehta J. Nama K. S. Barot M. Saharan B. S. (2024). Exploring fungal endophytes in a medicinal plant, lavandula officinalis L.: isolation, characterization, and plant growth-promoting potential. Ann. Phytomed. 13, 1–9. doi: 10.54085/ap.2024.13.2.101

[B5] Abdusamatov S. Yasenjiang A. Mamarakhimov O. Kuziyev S. Kudaybergenova U. Kholboeva G. (2025b). “Isolation and characterization of *Lycium barbarum* endophytic bacteria,” in BIO Web of Conferences, (, Les Ulis: EDP Sciences), 01013. doi: 10.1051/bioconf/202517301013

[B6] Afrin S. Bhuiyan M. N. I. (2023). Antagonistic activity of *Bacillus amyloliquefaciens* subsp. amyloliquefaciens against multidrug resistant *Serratia rubidaea*. Curr. Res. Microb. Sci. 5:100206. doi: 10.1016/j.crmicr.2023.100206 38089002 PMC10711391

[B7] Afzal I. Shinwari Z. K. Sikandar S. Shahzad S. (2019). Plant beneficial endophytic bacteria: mechanisms, diversity, host range and genetic determinants. Microbiol. Res. 221, 36–49. doi: 10.1016/j.micres.2019.02.001 30825940

[B8] Aremu A. O. Omogbene T. O. Fadiji T. Lawal I. O. Opara U. L. Fawole O. A. (2024). Plants as an alternative to the use of chemicals for crop protection against biotic threats: trends and future perspectives. Eur. J. Plant Pathol. 170, 711–766. doi: 10.1007/s10658-024-02924-y

[B9] Baltruschat H. Hummel J. Gillmeister M. Ratering S. Kabrodt K. Sieverding E. . (2025). Multifunctional endophytic bacteria intimately associated within spores of arbuscular mycorrhizal fungi in a chernozem soil in Central Europe. Eur. J. Soil Biol. 126:103760. doi: 10.1016/j.ejsobi.2025.103760

[B10] Biswas S. Philip I. Jayaram S. Sarojini S. (2023). Endophytic bacteria Klebsiella spp. and Bacillus spp. from Alternanthera philoxeroides in Madiwala Lake exhibit additive plant growth-promoting and biocontrol activities. J. Genet. Eng. Biotechnol. 21:153. doi: 10.1186/s43141-023-00620-8 38030944 PMC10686955

[B11] Bradford M. M. (1976). A rapid and sensitive method for the quantitation of microgram quantities of protein utilizing the principle of protein-dye binding. Anal. Biochem. 72, 248–254. doi: 10.1016/0003-2697(76)90527-3 942051

[B12] Corral-Federico A. G. Meza-Contreras J. J. Delgado-Ramírez C. S. Hernández-Martínez R. Méndez-Bravo A. Méndez-Bravo A. . (2024). Colonization of solanaceous crops by endophytic and rhizospheric plant growth-promoting bacteria from the native Solanaceae *Solanum hindsianum* Benth. Microbe 3:100089. doi: 10.1016/j.microb.2024.100089

[B13] de Lima J. D. Monteiro P. H. R. Rivadavea W. R. Barbosa M. Cordeiro R. D. Garboggini F. F. . (2024). Potential of endophytic bacteria from Acacia mearnsii: Phosphate solubilization, indole acetic acid production, and application in wheat. Appl. Soil Ecol. 196:105315. doi: 10.1016/j.apsoil.2024.105315

[B14] de-Bashan L. Nannipieri P. (2024). Recommendations for plant growth-promoting bacteria inoculation studies. Biol. Fertil. Soils 60, 259–261. doi: 10.1007/s00374-024-01798-w

[B15] Djalolova B. Abdusamatov S. Shurigin V. Jabborova D. Turaeva B. Kutlieva G. . (2024). Bacteria associated with grapevine (*Vitis vinifera* L.) rhizosphere and their efficacy in plant growth promotion. Plant Sci. 11, 1739–1746.

[B16] Drożdżyński P. Rutkowska N. Rodziewicz M. Marchut-Mikołajczyk O. (2024). Bioactive compounds produced by endophytic bacteria and their plant hosts—an insight into the world of chosen herbaceous ruderal plants in Central Europe. Molecules 29:4456. doi: 10.3390/molecules29184456 39339451 PMC11433698

[B17] Duhan P. Bansal P. Rani S. (2020). Isolation, identification and characterization of endophytic bacteria from medicinal plant Tinospora cordifolia. S. Afr. J. Bot. 134, 43–49. doi: 10.1016/j.sajb.2020.01.047

[B18] Finkel O. M. Castrillo G. Herrera Paredes S. Salas González I. Dangl J. L. (2017). Understanding and exploiting plant beneficial microbes. Curr. Opin. Plant Biol. 38, 155–163. doi: 10.1016/j.pbi.2017.04.018 28622659 PMC5561662

[B19] Frans E. N. Kwembeya E. Sibanda T. Uzabakiriho J. D. (2025). Plant growth-promoting traits of rhizosphere and endophytic bacteria associated with *myrothamnus flabellifolius* (WELW). S. Afr. J. Bot. 183, 1–12. doi: 10.1016/j.sajb.2025.05.015

[B20] Gad A. M. Suleiman W. B. El-Sheikh H. H. Elmezayen H. A. Beltagy E. A. (2022). Characterization of cellulase from Geotrichum candidum strain Gad1 approaching bioethanol production. Arab. J. Sci. Eng. 47, 6837–6850. doi: 10.1007/s13369-021-06391-z

[B21] Gayathri R. S. Sheeba M. S. Chandran S. S. John S. Chiseena C. T. John S. . (2025). Isolation, identification and bioprospecting potential of *Bacillus subtilis*, endophytic bacterium from *Bruguiera gymnorrhiza* (L.) Lam. ex Savigny. Microb. Pathog. 203:107458. doi: 10.1016/j.micpath.2025.107458 40058417

[B22] Grobelak A. Kokot P. Swiatek J. Jaskulak M. Rorat A. (2018). Bacterial ACC deaminase activity in promoting plant growth on areas contaminated with heavy metals. J. Ecol. Eng. 19:89818. doi: 10.12911/22998993/89818

[B23] Gupta G. Paul S. Singh S. Pietramellara G. Pathan S. I. Danish S. . (2022). Exploring functional diversity and community structure of diazotrophic endophytic bacteria associated with *Pennisetum glaucum* growing under field in a semi-arid region. Land 11:991. doi: 10.3390/land11070991

[B24] Gupta R. Kumar R. Al-Qahtani W. H. Abdel-Maksoud M. A. (2024). Exploring the uncharted: Zinc and phosphate solubilization in Zn-P isolates from wheat rhizosphere inceptisols. J. King Saud Univ. Sci. 36:103509. doi: 10.1016/j.jksus.2024.103509

[B25] Hardoim P. R. van Overbeek L. S. van Elsas J. D. (2008). Properties of bacterial endophytes and their proposed role in plant growth. Trends Microbiol. 16, 463–471. doi: 10.1016/j.tim.2008.07.008 18789693

[B26] Jabborova D. Bozorov T. Li L. Kistaubayeva A. Egamberdieva D. (2021a). Identification and characterization of endophytic bacteria isolated from root nodules of lentil (*Lens culinaris* L.) grown on saline soils. Int. J. Biol. Chem. 14, 39–46. doi: 10.26577/ijbch.2021.v14.i2.05

[B27] Jabborova D. Davranov K. Jabbarov Z. Bhowmik S. N. Ercisli S. Danish S. . (2022). Dual inoculation of plant growth-promoting Bacillus endophyticus and Funneliformis mosseae improves plant growth and soil properties in ginger. Acs Omega. 7, 34779–34788. doi: 10.1021/acsomega.2c02353 36211029 PMC9535732

[B28] Jabborova D. Davranov K. Jabbarov Z. Enakiev Y. Abdrakhmanov T. Datta R. . (2025a). Impact of growth-promoting Endophytic Bacteria on Ginger Plant growth. Indian J. Microbiol. 65, 991–1000. doi: 10.1007/s12088-024-01379-3 PMC12246303 40655335

[B29] Jabborova D. Enakiev Y. Sulaymanov K. Kadirova D. Ali A. Annapurna K. (2021b). Plant growth promoting bacteria *Bacillus subtilis* promote growth and physiological parameters of *Zingiber officinale Roscoe*. Plant Sci. Today 8, 66–71. doi: 10.14719/pst.2021.8.1.997

[B30] Jabborova D. Kadirova D. Jabbarov Z. Thakker J. N. Rathod K. Jabborov M. . (2025b). Impact of plant growth promoting rhizobacteria inoculation on growth and physiological traits of ginger in field conditions. Antonie van Leeuwenhoek 118, 1–5. doi: 10.1007/s10482-025-02161-1 40892082

[B31] Kaur M. Karnwal A. (2023). Screening of plant growth-promoting attributes bearing endogenous bacteria from abiotic stress resisting high altitude plants. J. Agric. Food Res. 11:100489. doi: 10.1016/j.jafr.2022.100489

[B32] Khasanov S. Kulmatov R. Li F. van Amstel A. Bartholomeus H. Aslanov I. . (2023). Impact assessment of soil salinity on crop production in Uzbekistan and its global significance. Agric. Ecosyst. Environ. 342:108262. doi: 10.1016/j.agee.2022.108262

[B33] Kim S. H. Kim W. J. Ryu J. Yerefu Y. Tesfaw A. (2025). Amylase Production by the New Strains of *Kocuria rosea* and *Micrococcus endophyticus* Isolated from Soil in the Guassa Community Conservation Area. Fermentation 11:211. doi: 10.3390/fermentation11040211

[B34] Kirchhoff L. Kirschbaum A. Joshi J. Bossdorf O. Scheepens J. F. Heinze J. (2019). Plant-soil feedbacks of *Plantago lanceolata* in the field depend on plant origin and herbivory. Front. Ecol. Evol. 7:422. doi: 10.3389/fevo.2019.00422

[B35] Krimi Z. Alim D. Djellout H. Tafifet L. Mohamed-Mahmoud F. Raio A. (2016). Bacterial endophytes of weeds are effective biocontrol agents of *Agrobacterium* spp., *Pectobacterium* spp., and promote growth of tomato plants. *Phytopathol. Mediterr*. 184–196.

[B36] Kumar A. Singh R. Yadav A. Giri D. D. Singh P. K. Pandey K. D. (2016). Isolation and characterization of bacterial endophytes of *Curcuma longa* L. 3 Biotech 6:60. doi: 10.1007/s13205-016-0393-y PMC4752947 28330130

[B37] Laanet P. R. Bragina O. Jõul P. Vaher M. (2024). *Plantago major* and *plantago lanceolata* exhibit antioxidant and borrelia burgdorferi inhibiting activities. Int. J. Mol. Sci. 25:7112. doi: 10.3390/ijms25137112 39000214 PMC11240987

[B38] Lane D. J. (1991). “16S/23S rRNA sequencing„” in Nucleic Acid Techniques in Bacterial Systematic, eds. E. Stackebrandt and M. Goodfellow (New York, NY: John Wiley and Sons), 115–175.

[B39] Li Z. Huang L. Chen X. Liu Q. Liu Y. Liu C. . (2025). Contribution of plant growth-promoting endophytic bacteria from hyperaccumulator to non-host plant zinc nutrition and health. Int. J. Phytoremediation 27, 23–35. doi: 10.1080/15226514.2024.2395983 39185733

[B40] Lovecká P. Kroneislová G. Novotná Z. Röderová J. Demnerová K. (2023). Plant growth-promoting endophytic bacteria isolated from *Miscanthus giganteus* and their antifungal activity. Microorganisms 11:2710. doi: 10.3390/microorganisms11112710 38004722 PMC10672898

[B41] Mamarasulov B. Davranov K. Jahan M. S. Jabborova D. Nasif O. Ansari M. J. . (2022). Characterization, enzymatic and biochemical properties of endophytic bacterial strains of the medicinal plant *Ajuga turkestanica* (Rgl.) Brig (*Lamiaceae*). *J. King Saud Univ. Sci*. 34:102183. doi: 10.1016/j.jksus.2022.102183

[B42] McKinnon A. C. (2016). “Plant tissue preparation for the detection of an endophytic fungus in planta,” in Microbial-Based Biopesticides: Methods and Protocols (New York, NY: Springer), 167–173. 10.1007/978-1-4939-6367-6_13 27565499

[B43] Muromtsev G. S. Nestyuk M. N. (1960). Quantitative colorimetric determination of gibberellic acid. Bull. Agric. Sci. 2, 119–122.

[B44] Pal G. Saxena S. Kumar K. Verma A. Kumar D. Shukla P. . (2024). Seed endophytic bacterium *Lysinibacillus* sp.(ZM1) from maize (*Zea mays* L.) shapes its root architecture through modulation of auxin biosynthesis and nitrogen metabolism. Plant Physiol. Biochem. 212:108731. doi: 10.1016/j.plaphy.2024.108731 38761545

[B45] Panigrahi S. Mohanty S. Rath C. C. (2020). Characterization of endophytic bacteria *Enterobacter cloacae* MG00145 isolated from *Ocimum sanctum* with indole acetic acid (IAA) production and plant growth promoting capabilities against selected crops. S. Afr. J. Bot. 134, 17–26. doi: 10.1016/j.sajb.2019.09.017

[B46] Penczykowski R. M. Anna-Liisa L. Britt K. (2016).Understanding the ecology and evolution of host–parasite interactions across scales. Evol. Appl. 9, 37–52. doi: 10.1111/eva.12294 27087838 PMC4780374

[B47] Penrose D. M. Glick B. R. (2003). Methods for isolating and characterizing ACC deaminase-containing plant growth-promoting rhizobacteria. Physiol. Plant. 118, 10–15. doi: 10.1034/j.1399-3054.2003.00086.x 12702008

[B48] Rahamouz-Haghighi S. Kh B. Mohsen-Pour N. Sharafi A. (2022). In vitro evaluation of cytotoxicity and antibacterial activities of ribwort plantain (plantago lanceolata L.) root fractions and phytochemical analysis by gas chromatography-mass spectrometry. *Arch. Razi Inst*. 77:2131. 10.22092/ARI.2022.358045.2143 PMC10237569 37274901

[B49] Ramírez C. Cardozo M. Gastón M. L. Galdeano E. Collavino M. M. (2024). Plant growth promoting activities of endophytic bacteria from *Melia azedarach* (Meliaceae) and their influence on plant growth under gnotobiotic conditions. Heliyon 10:e35814. doi: 10.1016/j.heliyon.2024.e35814 39170558 PMC11337034

[B50] Rani S. Kumar P. Dahiya P. Maheshwari R. Dang A. S. Suneja P. (2022). Endophytism: a multidimensional approach to plant–prokaryotic microbe interaction. Front. Microbiol. 13:861235. doi: 10.3389/fmicb.2022.861235 35633681 PMC9135327

[B51] Riseh R. S. Vatankhah M. Hassanisaadi M. Ait Barka E. (2024). Unveiling the role of hydrolytic enzymes from soil biocontrol bacteria in sustainable phytopathogen management. Front. Biosci. Landmark 29:105. doi: 10.31083/j.fbl2903105 38538262

[B52] Rosini B. Bulla A. M. Polonio J. C. Polli A. D. da Silva A. A. Schoffen R. P. . (2025). Isolation, identification, and bioprospection of endophytic bacteria from medicinal plant *Mikania glomerata* (Spreng.) and the consortium of *Pseudomonas* as plant growth promoters. Biocatal. Agric. Biotechnol. 64:103530. doi: 10.1016/j.bcab.2025.103530

[B53] Samuelsen A. B. (2000). The traditional uses, chemical constituents and biological activities of *Plantago major* L. A review. J. Ethnopharmacol. 71, 1–21. doi: 10.1016/S0378-8741(00)00212-9 10904143 PMC7142308

[B54] Santoyo G. Guzmán-Guzmán P. Parra-Cota F. I. Santos-Villalobos S. D. L. Orozco-Mosqueda M. D. C. Glick B. R. (2021). Plant growth stimulation by microbial consortia. Agronomy 11:219. doi: 10.3390/agronomy11020219

[B55] Santoyo G. Moreno-Hagelsieb G. del Carmen Orozco-Mosqueda M. Glick B. R. (2016). Plant growth-promoting bacterial endophytes. Microbiol. Res. 183, 92–99. doi: 10.1016/j.micres.2015.11.008 26805622

[B56] Sarvepalli M. Velidandi A. Korrapati N. (2023). Optimization of siderophore production in three marine bacterial isolates along with their heavy-metal chelation and seed germination potential determination. Microorganisms 11:2873. doi: 10.3390/microorganisms11122873 38138017 PMC10746010

[B57] Shahid M. Singh U. B. Khan M. S. Singh P. Kumar R. Singh R. N. . (2023). Bacterial ACC deaminase: insights into enzymology, biochemistry, genetics, and potential role in amelioration of environmental stress in crop plants. Front. Microbiol. 14:1132770. doi: 10.3389/fmicb.2023.1132770 37180266 PMC10174264

[B58] Sharma N. (2024). Endophytic bacteria associated with critically endangered medicinal plant *Trillium govanianum* (Wall ex. Royle) and their potential in soil nutrition alleviation. Plant Stress 11:100349. doi: 10.1016/j.stress.2024.100349

[B59] Sherzad Z. Nawakht N. A. Sherzad F. (2025). Plant growth-promoting endophytic bacteria: a sustainable solution for climate change and environmental stresses in agriculture. Discov Appl. Sci. 7:894. doi: 10.1007/s42452-025-07123-w

[B60] Shreshtha K. Raj S. Pal A. K. Tripathi P. Choudhary K. K. Mitra D. . (2024). Isolation and identification of rhizospheric and endophytic bacteria from cucumber plants irrigated with wastewater: exploring their roles in plant growth promotion and disease suppression. Curr. Res. Microb. Sci. 7:100256. doi: 10.1016/j.crmicr.2024.100256 39717060 PMC11665314

[B61] Souza Y. P. A. Schloter M. Weisser W. Huang Y. Schulz S. (2024). The seeds of *Plantago lanceolata* comprise a stable core microbiome along a plant richness gradient. Environ. Microbiome. 19:11. doi: 10.1186/s40793-024-00552-x 38308354 PMC10835927

[B62] Sriwati R. Maulidia V. Intan N. Oktarina H. Khairan K. Skala L. . (2023). Endophytic bacteria as biological agents to control fusarium wilt disease and promote tomato plant growth. Physiol. Mol. Plant Pathol. 125:101994. doi: 10.1016/j.pmpp.2023.101994

[B63] Tang R. Tian Q. Liu S. Gong Y. Li Q. Chen R. . (2025). Endophytic bacteria in different tissue compartments of African wild rice (*Oryza longistaminata*) promote perennial rice growth. J. Integr. Agric. 24, 1001–1016. doi: 10.1016/j.jia.2023.11.031

[B64] Thomloudi E. E. Tsalgatidou P. C. Baira E. Papadimitriou K. Venieraki A. Katinakis P. (2021). Genomic and metabolomic insights into secondary metabolites of the novel *Bacillus halotolerans* Hil4, an endophyte with promising antagonistic activity against gray mold and plant growth promoting potential. Microorganisms 9:2508. doi: 10.3390/microorganisms9122508 34946110 PMC8704346

[B65] Vanhee M. Floré K. Vanthourenhout S. Hellemans J. Muyldermans A. Reynders M. (2024). Implementation of full-length 16S nanopore sequencing for bacterial identification in a clinical diagnostic setting. Diagn. Microbiol. Infect. Dis. 108:116156. doi: 10.1016/j.diagmicrobio.2023.116156 38061217

[B66] Vendan R. T. Yu Y. J. Lee S. H. Rhee Y. H. (2010). Diversity of endophytic bacteria in ginseng and their potential for plant growth promotion. J. Microbiol. 48, 559–565. doi: 10.1007/s12275-010-0082-1 21046332

[B67] Vimal S. R. Singh J. S. Prasad S. M. (2025). Prospective of indole-3-acteic acid (IAA) and endophytic microbe *Bacillus subtilis* strain SSA4 in paddy seedlings development and ascorbate–glutathione (AsA-GSH) cycle regulation to mitigate NaCl toxicity. Mol. Biotechnol. 67, 3054–3069. doi: 10.1007/s12033-023-00743-w 37087717

[B68] Wang Y. Zhang Y. Cong H. Li C. Wu J. Li L. . (2023). Cultivable endophyte resources in medicinal plants and effects on hosts. Life 13:1695. doi: 10.3390/life13081695 37629552 PMC10455732

[B69] Woo J. I. Injamum-Ul-Hoque M. Zainurin N. Shaffique S. Kwon E. H. Gam H. J. . (2023). Gibberellin-producing bacteria isolated from coastal soil enhance seed germination of mallow and broccoli plants under saline conditions. BioTech 12:66. doi: 10.3390/biotech12040066 38131678 PMC10741878

[B70] Xie X. H. Fu X. Yan X. Y. Peng W. F. Kang L. X. (2021). A broad-specificity chitinase from *Penicillium oxalicum* k10 exhibits antifungal activity and biodegradation properties of chitin. Mar. Drugs 19:356. doi: 10.3390/md19070356 34201595 PMC8307900

[B71] Younis T. Rahman S. Rahman L. Iqrar I. Shinwari Z. K. (2024). Exploring the impact of endophytic bacteria on mitigating salinity stress in *Solanum lycopersicum* L. Plant Stress 12:100467. doi: 10.1016/j.stress.2024.100467

[B72] Zhakipbekov K. Turgumbayeva A. Issayeva R. Kipchakbayeva A. Kadyrbayeva G. Tleubayeva M. . (2023). Antimicrobial and other biomedical properties of extracts from *Plantago major, Plantaginaceae. Pharmaceuticals* 16:1092. doi: 10.3390/ph16081092 PMC10458736 37631007

